# Cumulative incidence and risk of infection in patients with rheumatoid arthritis treated with janus kinase inhibitors: A systematic review and meta-analysis

**DOI:** 10.1371/journal.pone.0306548

**Published:** 2024-07-31

**Authors:** Konstantinos Ouranos, Diana V. Avila, Evangelia K. Mylona, Athanasios Vassilopoulos, Stephanos Vassilopoulos, Fadi Shehadeh, Eleftherios Mylonakis

**Affiliations:** 1 Department of Medicine, Houston Methodist Research Institute, Houston, TX, United States of America; 2 Department of Medicine, Warren Alpert Medical School of Brown University, Rhode Island Hospital, Providence, RI, United States of America; 3 School of Electrical and Computer Engineering, National Technical University of Athens, Athens, Greece; 4 Department of Medicine, Weill Cornell Medical College, New York, NY, United States of America; Children’s National Hospital, George Washington University, UNITED STATES

## Abstract

Patients with rheumatoid arthritis (RA) who receive immunosuppressive medications have a heightened risk of infection. The goal of our study was to calculate the pooled cumulative incidence and risk of infection in patients with RA treated with Janus kinase inhibitors (JAKi). The PubMed and EMBASE databases were queried for randomized controlled trials comparing patients with RA treated with JAKi (upadacitinib, baricitinib, tofacitinib, peficitinib, or filgotinib), defined as the treatment group, compared with control subjects, defined as participants receiving placebo or treatment regimen that was similar to that of participants in the treatment group, with the exception of JAKi. The primary study endpoint was the relative risk (RR) of any-grade and severe infection. The secondary endpoints were RR and cumulative incidence of opportunistic infections, herpes zoster, and pneumonia. The Stata v17 software was used for all data analysis. Results showed that treatment with baricitinib was associated with an increased risk of any-grade (RR 1.34; 95% CI: 1.19–1.52) and opportunistic (RR 2.69; 95% CI: 1.22–5.94) infection, whereas treatment with filgotinib (RR 1.21; 95% CI: 1.05–1.39), peficitinib (RR 1.40; 95% CI: 1.05–1.86) and upadacitinib (RR 1.30; 95% CI: 1.09–1.56) was associated with increased risk of any-grade infection only. Analysis based on type of infection showed a pooled cumulative incidence of 32.44% for any-grade infections, 2.02% for severe infections, 1.74% for opportunistic infections, 1.56% for herpes zoster, and 0.49% for pneumonia in patients treated with any JAKi during the follow-up period. Treatment with specific JAKi in patients with RA is associated with an increased risk of any-grade and opportunistic infections but not severe infection. Close clinical monitoring of patients with RA treated with JAKi is required to establish the long-term infection risk profile of these agents.

## 1. Introduction

Disease-modifying antirheumatic drugs (DMARDs) are currently the first-line treatment option for patients with rheumatoid arthritis (RA) [1]. Among the available DMARDs, conventional synthetic DMARDs, including methotrexate and leflunomide, are first-line agents due to their proven efficacy and favorable safety profile [2]. In patients with inadequate response or contraindications to conventional synthetic DMARD monotherapy, treatment escalation to targeted synthetic DMARDs, such as biologic DMARDs and Janus kinase (JAK) inhibitors (JAKi), is recommended, either alone or in combination with conventional synthetic DMARDs [3].

Landmark phase III clinical trials have established the effectiveness of JAKi compared with placebo in methotrexate-naïve patients or patients with inadequate response to methotrexate or other DMARDs [4]. Tofacitinib, baricitinib, and upadacitinib are currently approved by the Food and Drug Administration for use in patients with RA. Each approved JAKi has varying specificity for different JAK isoforms, thus impacting distinct downstream pathways involved in the pathophysiology of RA [5].

Patients with RA are at increased risk of infection due to disease pathophysiology and the use of immunomodulatory therapy [6]. The use of JAKi has also been associated with an increased risk of infection, including opportunistic infections [7]. The infection risk profile of these agents has been attributed to the blockade of cytokines that use the JAK-Signal Transduction and Activator of Transcription (STAT) signaling pathway and are the primary drivers of host cellular and humoral immune responses against infection [8].

Although JAKi have been associated with a heightened risk of infection, their risk profile in patients with RA is not thoroughly described. The goal of our systematic review and meta-analysis was to estimate the pooled cumulative incidence and infection risk in patients with RA receiving JAKi compared with patients with RA receiving placebo or treatment regimen that was similar with that of participants in the treatment group, with the exception of JAKi.

## 2. Materials and methods

We used the Preferred Reporting Items for Systematic Reviews and Meta-Analyses (PRISMA) 2020 checklist [9] to perform this systematic review and meta-analysis, and we registered the study on PROSPERO (CRD42024507846).

### 2.1 Data sources and search strategies

PubMed and EMBASE databases were queried for studies in English, spanning from database inception to October 30, 2023, using the following search terms: (“rheumatoid arthritis” AND (tofacitinib OR baricitinib OR upadacitinib OR peficitinib OR filgotinib OR “JAK inhibitor*”). In order to find additional articles that would meet our inclusion criteria, we also manually checked the reference lists of extracted articles, as well as previously published meta-analyses, to consistently include all eligible papers in our manuscript.

### 2.2 Criteria for study inclusion in the analysis

We considered randomized controlled trials for inclusion in the analysis if they met the following criteria: a) Randomization of study participants to a treatment or control group occurred; b) Participants in the treatment group had a diagnosis of RA and received JAKi; c) Participants in the control group had a diagnosis of RA and received placebo or treatment regimen that was similar with that of participants in the treatment group, with the exception of JAKi; d) The diagnosis of RA was based on the 1987 American College of Rheumatology (ACR) Revised Criteria [10] or the ACR/European League Against Rheumatism (EULAR) 2010 classification criteria [11]; and e) included studies had available data for at least one of our pre-defined outcomes. Studies in which patients with RA were receiving concurrent therapy with agents other than JAKi were also included in the analysis. We excluded studies that were not randomized controlled trials, including case-control and/or cohort studies, review articles, and case reports. An abstract presentation was considered for inclusion in our analysis only if the results were not already published in full text manuscripts.

### 2.3 Study endpoints

Our primary study endpoint was to measure the relative risk (RR) of any-grade and severe infection in patients with RA receiving JAKi (treatment group), as compared with patients with RA receiving placebo or treatment regimen that was similar with that of participants in the treatment group, with the exception of JAKi (control group).

Our secondary study endpoints were the pooled cumulative incidence of any-grade and severe infection during two study periods in each included study: the first spanned the period from study initiation until primary outcome assessment, and the second was the follow-up period, extending beyond the primary outcome assessment until the end of each study. After primary outcome assessment, significant crossover between treatment and control groups occurred. As such, pooled cumulative incidences of infection were calculated at two separate periods to account for the differences in study population under examination.

Next, we measured the cumulative incidence and risk of opportunistic infections, including herpes zoster infection, in the treatment group compared with the control group. We harnessed the definitions proposed by Winthrop *et al*. [12] to calculate the number of opportunistic infections in our analysis. Lastly, we aimed to measure the RR and pooled cumulative incidence of different types of infections reported in the included studies. Such an analysis was carried out only when data were of sufficient quantity to run these analyses, in the treatment group compared with the control group. We used the National Cancer Institute’s Common Terminology Criteria for Adverse Events [13] to assign the grade of infectious complications.

### 2.4 Extraction of data and assessment of quality of included studies

Two reviewers (K.O. and D.A.) conducted an independent evaluation of study eligibility by reviewing titles and abstracts, and then conducted a full text review of the chosen studies. Data extracted from the selected studies included information regarding study design, demographic characteristics of study participants, medication history in addition to the use of JAKi, the specific JAKi used, information regarding primary and secondary outcome assessments, and infection-attributed mortality rate.

We independently evaluated (K.O. and D.A.) the included studies’ quality by harnessing the revised Cochrane risk-of-bias assessment tool for randomized trials (RoB 2) [14]. This tool assesses study quality against five domains: process of randomization, deviations from intended interventions, missing outcome data, outcome measurement, and selection of the reported results. Each individual study and all outcomes within those studies were categorized as having low risk, some concerns, or high risk of bias.

### 2.5 Statistical analysis

Random-effects meta-analysis using the restricted maximum likelihood method [15] was implemented to estimate the RR of infections in patients with RA in the treatment group compared with the controls. Additionally, we measured the pooled cumulative incidence of infections during the two study periods under consideration, as described above, and we harnessed the Freeman-Tukey double-arcsine transformation to stabilize the variances [16, 17]. We reported study outcomes using 95% confidence intervals (CI). We also used the *I*^*2*^ statistic to determine study heterogeneity. Low, moderate, and high heterogeneity was defined as an *I*^*2*^ value of 25, 50, and 75%, respectively [18]. In order to assess for publication bias and small study effects, the Egger’s test was used. [19]. For all data analysis and interpretation, we set statistical significance at α = 0.05.

In order to account for multiple extracted variables that could confound the association between JAKi administration and infection risk, we conducted a meta-regression analysis [20] by taking into consideration the following variables: mean age of patients’ in the intervention and control group, percentage of female study participants in each group, and concurrent use of methotrexate and/or glucocorticoids.

To perform all data analysis, we used the Stata v17 (Stata Corporation, College Station, TX) software.

## 3. Results

### 3.1 Database search results

**Fig 1:**
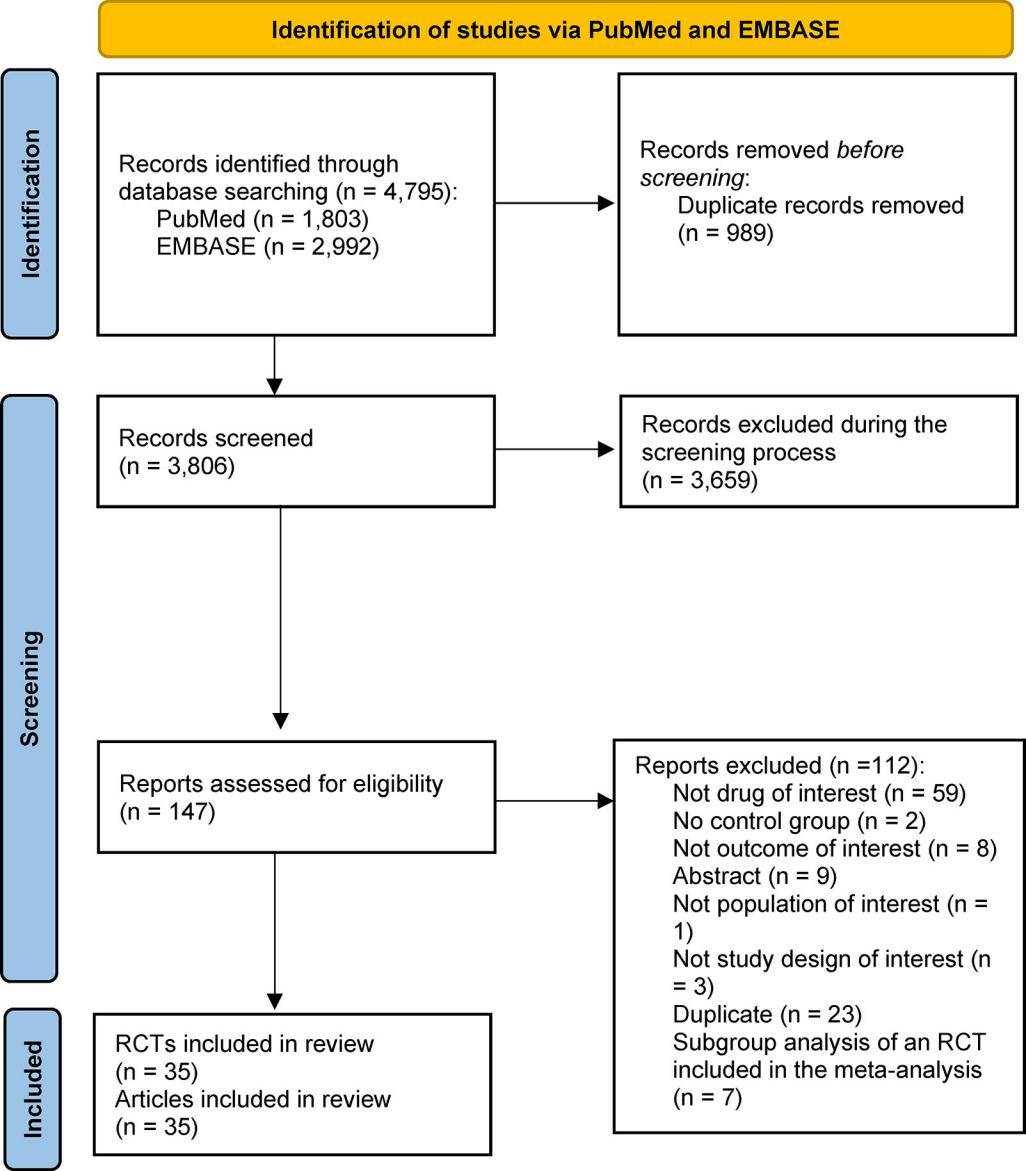
Flowchart outlining the selection process for studies included in the analysis.

We found 4,795 studies in our literature search in PubMed and EMBASE. We removed 989 duplicates and assessed 3,806 studies against our inclusion criteria. A total of 3,659 studies were excluded after careful review of their title and abstract, and, as such, we reviewed 147 publications in full text. We included 35 randomized controlled trials from 35 publications for the quantitative synthesis of the study. Details regarding the number of studies retrieved from each database, and reasons for excluding ineligible studies are presented in **Fig 1**.

### 3.2 Baseline characteristics of included studies and overview of study results

Out of the 35 studies, eight examined upadacitinib [21–28], seven examined baricitinib [29–35], five examined filgotinib [36–40], ten examined tofacitinib [41–50], and five examined peficitinib [51–55]. **S1 Table** displays the characteristics of the studies analyzed.

The included randomized controlled trials included data on 10,730 patients with RA in the treatment group and 4,628 patients with RA in the control group. Mean age of the patients in the treatment and control groups was 53.6 and 53.1 years, respectively. Additionally, 8,853 (82.5%) patients in the treatment group and 3,551 (76.7%) patients in the control group were female. Data regarding concomitant methotrexate use at study entry and for the duration of the study were available in 9,512 patients in the treatment group and 4,153 patients in the control group, of whom 7,975 (74.3%) and 3,623 (78.3%), respectively, were receiving methotrexate. Data regarding concomitant corticosteroid use at study entry and for the duration of the study were available in 8,226 patients in the treatment group and 3,732 patients in the control group, of whom 4,224 (51.3%) and 1,948 (52.2%), respectively, were receiving glucocorticoids. Overall, analysis based on type of infection during the period from study initiation until the time of primary outcome assessment for each study revealed that 2,213 out of 10,519 (16.70%) patients in the intervention arm developed any-grade infection, 133 out of 10,730 (1.24%) patients developed severe infection, and 115 out of 10,519 (1.09%) patients developed opportunistic infections (**S2 Table**). During follow-up, 1,880 out of 6,835 (27.51%) patients developed any-grade infection, 143 out of 6,835 (2.09%) patients developed severe infection, and 132 out of 6,835 (1.93%) patients developed opportunistic infections (**S3 Table**).

### 3.3 Primary and secondary study endpoints

#### 3.3.1 Risk of any-grade infection in patients with RA receiving JAKi compared with control subjects

**Fig 2:**
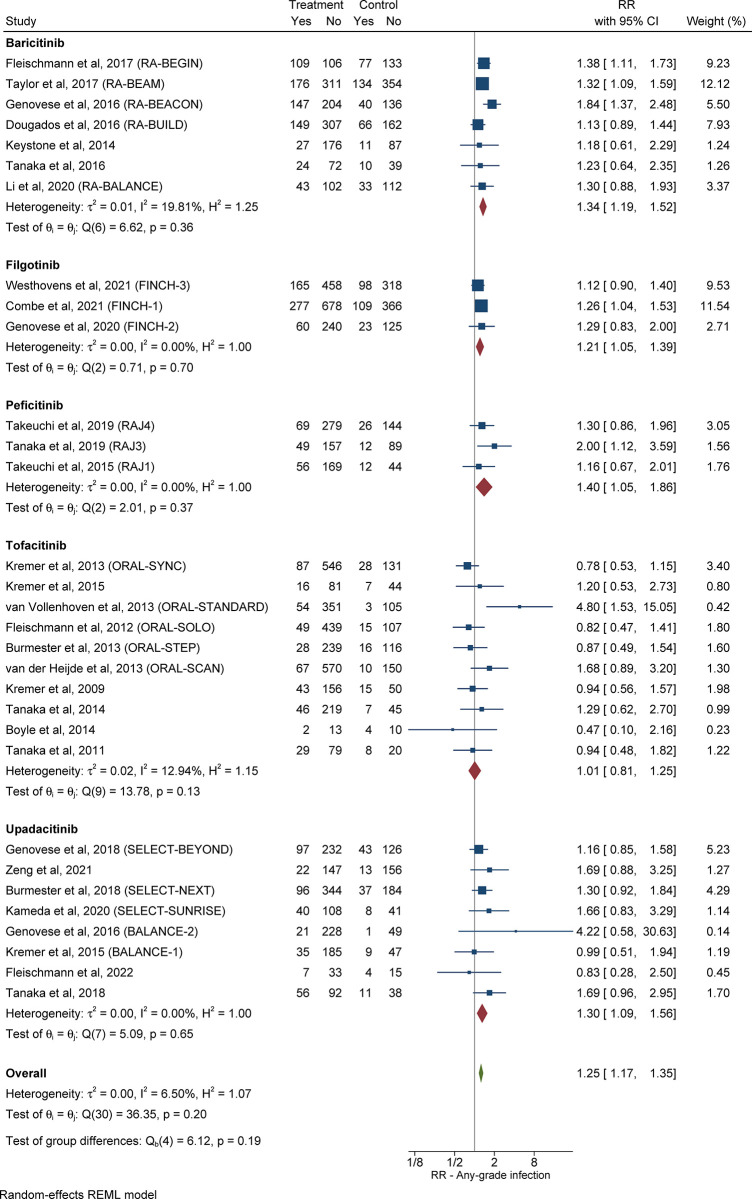
Relative risk of any-grade infection in patients with RA treated with JAKi compared with control subjects. The size of the square is representative of the weight assigned to the respective study for outcome analysis. The 95% CI for each study is represented by the horizontal lines. The diamond shape represents the pooled estimate of the analysis. Abbreviations: CI: confidence interval; JAKi: Janus-activated kinase inhibitor; RA: rheumatoid arthritis; RR: relative risk.

The overall RR of any-grade infection in patients with RA in the treatment group compared with the control group was 1.25 (95% CI: 1.17–1.35, *I*^*2*^ = 6.50%), as shown in **Table 1** and **Fig 2**. Out of eight studies [21–28] that assessed patients with RA who were treated with upadacitinib, 374 out of 1,743 patients (21.5%) in the treatment group and 126 out of 782 (16.1%) control subjects developed any-grade infection [RR 1.30 (95% CI: 1.09–1.56, *I*^*2*^ = 0.00%)].

**Table 1:** Risk of any-grade, severe, and opportunistic infections, herpes zoster, and pneumonia in patients with RA in the treatment group compared with control subjects.

Treatment agent	RR	95% CI	Heterogeneity, *I*^*2*^
*Any-grade infections*			
Upadacitinib	1.30	1.09–1.56	0.00%
Baricitinib	1.34	1.19–1.52	19.81%
Filgotinib	1.21	1.05–1.39	0.00%
Tofacitinib	1.01	0.81–1.25	12.94%
Peficitinib	1.40	1.05–1.86	0.00%
Any JAKi	1.25	1.17–1.35	6.50%
*Severe infections*			
Upadacitinib	2.05	0.76–5.55	0.00%
Baricitinib	0.90	0.54–1.51	0.00%
Filgotinib	1.19	0.64–2.19	0.00%
Tofacitinib	1.19	0.48–2.95	0.00%
Peficitinib	1.23	0.29–5.12	0.00%
Any JAKi	1.13	0.81–1.57	0.00%
*Opportunistic infections*			
Upadacitinib	1.41	0.64–3.10	0.00%
Baricitinib	2.69	1.22–5.94	0.00%
Filgotinib	1.18	0.52–2.71	0.00%
Tofacitinib	1.13	0.41–3.13	0.00%
Peficitinib	1.18	0.31–4.56	0.00%
Any JAKi	1.52	1.01–2.27	0.00%
*Herpes zoster*			
Upadacitinib	1.26	0.57–2.81	0.00%
Baricitinib	2.54	1.14–5.65	0.00%
Filgotinib	1.44	0.58–3.61	0.00%
Tofacitinib	1.13	0.41–3.13	0.00%
Peficitinib	0.95	0.24–3.77	0.00%
Any JAKi	1.50	0.99–2.27	0.00%
*Pneumonia*			
Upadacitinib	0.95	0.29–3.05	0.00%
Baricitinib	0.94	0.33–2.71	0.00%
Filgotinib	0.79	0.23–2.74	0.00%
Tofacitinib	0.61	0.21–1.80	0.00%
Peficitinib	0.71	0.12–4.06	0.00%
Any JAKi	0.80	0.47–1.37	0.00%

Abbreviations: CI: confidence interval; JAKi: Janus-activated kinase inhibitor; RA: rheumatoid arthritis; RR: relative risk.

Six studies [29–31, 33–35] assessed patients with RA who were treated with baricitinib, in which 675 out of 1,953 (34.6%) patients in the treatment group and 371 out of 1,394 (26.6%) control subjects developed any-grade infection [RR 1.34 (95% CI: 1.19–1.52, *I*^*2*^ = 19.81%)].

Three [36–38] studies assessed patients with RA who were treated with filgotinib, in which 502 out of 1,878 (26.7%) patients in the treatment group and 230 out of 1,039 (22.1%) control subjects developed any-grade infection [RR 1.21 (95% CI: 1.05–1.39, *I*^*2*^ = 0.00%)].

Out of ten studies [41–50] that assessed patients with RA who were treated with tofacitinib, 421 out of 3,114 (13.5%) patients in the treatment group and 113 out of 891 (12.7%) control subjects developed any-grade infection [RR 1.01 (95% CI: 0.81–1.25, *I*^*2*^ = 12.94%)].

Three studies [52–54] assessed peficitinib in patients with RA, in which 174 out of 779 (22.3%) patients in the treatment group and 50 out of 327 (15.3%) control subjects developed any-grade infection [RR 1.40 (95% CI: 1.05–1.86, *I*^*2*^ = 0.00%)].

#### 3.3.2 Pooled cumulative incidence of any-grade infection in patients with RA receiving JAKi

Among 9,975 patients with RA receiving JAKi, the pooled cumulative incidence of any-grade infection from study initiation until primary outcome assessment, over a mean duration of 18.2 weeks, was 21.59% (95% CI: 18.10%-25.71%, *I*^*2*^ = 94.6%) (**Table 2 and S1 Fig**). The pooled cumulative incidence of any-grade infection during follow-up, over a mean duration of 30.8 weeks, in 6,835 patients with RA treated with JAKi was 32.44% (95% CI: 21.04%-45.01%, *I*^*2*^ = 99.1%) (**S2 Fig**). The pooled cumulative incidence of any-grade infection from study initiation until primary outcome assessment, as well as during follow-up, for every JAKi under consideration, is presented in the **S1 File**

**Table 2:** Pooled cumulative incidence of any-grade, severe, and opportunistic infections, herpes zoster, and pneumonia in patients with RA treated with JAKi.

Treatment agent	Cumulative incidence	95% CI	Heterogeneity, *I*^*2*^	Study period (weeks)
**Period 1: Study initiation until primary outcome assessment**				
*Any-grade infections*				
Upadacitinib	20.71%	14.40%-27.80%	91.20%	12
Baricitinib	32.40%	24.40%-40.95%	93.30%	24.6
Filgotinib	21.91%	14.97%-29.76%	94.70%	31
Tofacitinib	14.40%	11.67%-17.36%	76.90%	11.6
Peficitinib	22.38%	19.22%-25.71%	NA**	12
Any JAKi	21.59%	18.10%-25.71%	94.6%	18.2
*Severe infections*				
Upadacitinib	0.99%	0.34%-1.90%	49.50%	12
Baricitinib	1.66%	1.00%-2.45%	26.30%	24.6
Filgotinib	1.54%	1.09%-2.07%	0.00%	27.2
Tofacitinib	0.41%	0.08%-0.92%	44.40%	11.6
Peficitinib	0.52%	0.15%-1.03%	0.00%	12
Any JAKi	0.92%	0.65%-1.23%	43.8%	17.5
*Opportunistic infections*				
Upadacitinib	1.50%	0.86%-2.29%	19.10%	12
Baricitinib	1.20%	0.49%-2.14%	56.10%	24.6
Filgotinib	0.86%	0.38%-1.51%	49.40%	28
Tofacitinib	0.25%	0.00%-0.87%	68.70%	11.6
Peficitinib	0.70%	0.20%-1.44%	35.20%	12
Any JAKi	0.82%	0.52%-1.44%	59.3%	17.6
*Herpes zoster*				
Upadacitinib	1.32%	0.72%-2.07%	18.40%	12
Baricitinib	1.13%	0.48%-2.00%	51.30%	24.6
Filgotinib	0.82%	0.39%-1.39%	38.70%	28
Tofacitinib	0.25%	0.00%-0.87%	68.70%	11.6
Peficitinib	0.54%	0.09%-1.25%	40.00%	12
Any JAKi	0.74%	0.09%-1.25%	40.0%	17.6
*Pneumonia*				
Upadacitinib	0.24%	0.00%-0.73%	30.20%	12
Baricitinib	0.26%	0.04%-0.60%	0.00%	24.6
Filgotinib	0.37%	0.03%-0.96%	NA*	29.3
Tofacitinib	0.00%	0.00%-0.11%	18.90%	12
Peficitinib	0.12%	0.00%-0.53%	0.00%	12
Any JAKi	0.11%	0.00%-0.23%	4.17%	18
**Period 2: Follow-up from primary outcome assessment until end of study**				
*Any-grade infections*				
Upadacitinib	67.74%	63.63%-71.72%	NA*	50
Baricitinib	38.33%	9.46%-72.76%	99.30%	27
Filgotinib	38.08%	35.32%-40.87%	NA*	20
Tofacitinib	13.15%	9.30%-17.56%	90.90%	16.8
Peficitinib	37.27%	34.18%-40.42%	NA*	40
Any JAKi	32.44%	21.04%-45.01%	99.1%	30.8
*Severe infections*				
Upadacitinib	7.85%	5.66%-10.36%	NA*	50
Baricitinib	1.95%	0.76%-3.61%	63.50%	27
Filgotinib	2.58%	1.73%-3.58%	NA*	20
Tofacitinib	1.05%	0.48%-1.82%	66.20%	16.8
Peficitinib	1.27%	0.62%-2.13%	NA*	40
Any JAKi	2.02%	1.22%-3.01%	84.3%	30.8
*Opportunistic infections*				
Upadacitinib	9.63%	7.21%-12.35%	NA*	50
Baricitinib	1.10%	0.00%-4.48%	91.90%	27
Filgotinib	0.87%	0.39%-1.52%	NA*	20
Tofacitinib	0.56%	0.04%-1.54%	85.00%	16.8
Peficitinib	3.39%	2.30%-4.67%	NA*	40
Any JAKi	1.74%	0.71%-3.16%	92.8%	30.8
*Herpes zoster*				
Upadacitinib	8.89%	6.56%-11.53%	NA*	50
Baricitinib	1.10%	0.00%-4.16%	90.60%	27
Filgotinib	0.87%	0.39%-1.52%	NA*	20
Tofacitinib	0.35%	0.00%-1.35%	88.30%	16.8
Peficitinib	3.27%	2.20%-4.54%	NA*	40
Any JAKi	1.56%	0.59%-2.92%	92.8%	30.8
*Pneumonia*				
Upadacitinib	2.37%	1.18%-3.91%	NA*	50
Baricitinib	0.48%	0.05%-1.21%	37.00%	27
Filgotinib	0.70%	0.27%-1.30%	NA*	20
Tofacitinib	0.12%	0.00%-0.34%	23.70%	16.8
Peficitinib	0.74%	0.25%-1.43%	NA*	40
Any JAKi	0.49%	0.20%-0.89%	69.1%	30.8

*Heterogeneity could not be reported due to an inadequate number of studies under investigation.

Abbreviations: CI: confidence interval; JAKi: Janus-activated kinase inhibitor; RA: rheumatoid arthritis; RR: relative risk.

#### 3.3.3 Relative risk of severe infection in patients with RA receiving JAKi compared with control subjects

**Fig 3:**
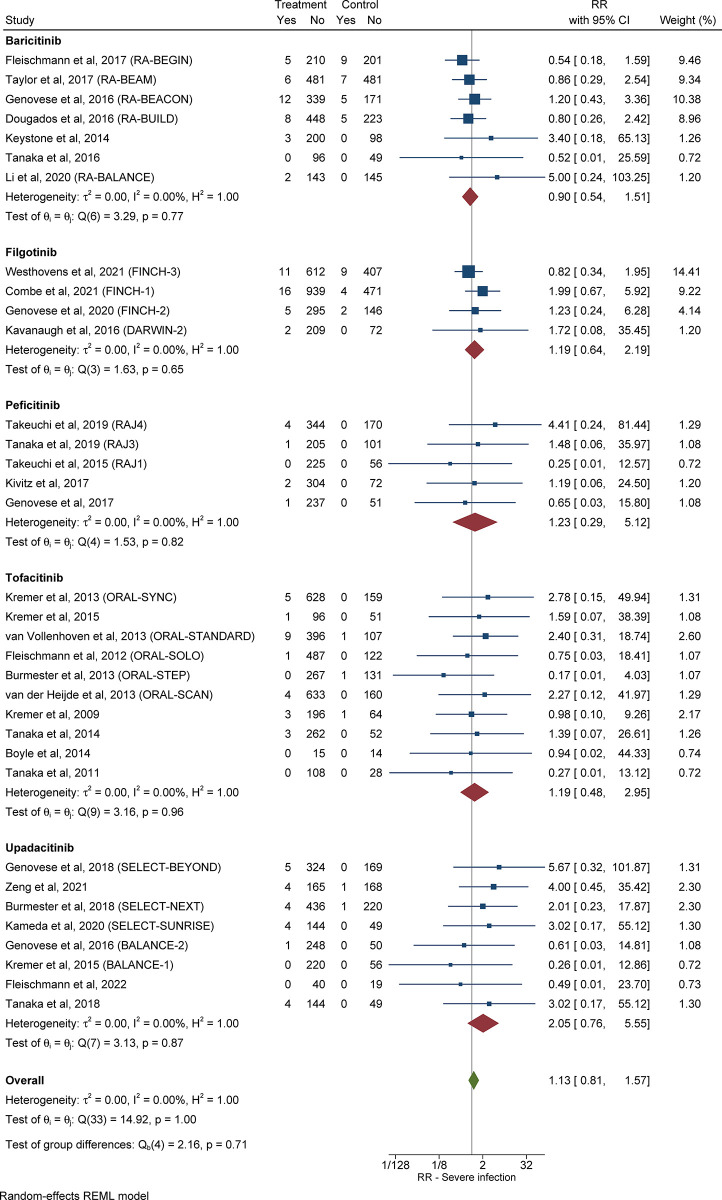
Relative risk of severe infection in patients with RA treated with JAKi compared with control subjects. The size of the square is representative of the weight assigned to the respective study for outcome analysis. The 95% CI for each study is represented by the horizontal lines. The diamond shape represents the pooled estimate of the analysis. Abbreviations: CI: confidence interval; JAKi: Janus-activated kinase inhibitor; RA: rheumatoid arthritis; RR: relative risk.

The overall RR for severe infection in patients with RA in the treatment group compared with control subjects was 1.13 (95% CI: 0.81–1.57, *I*^*2*^ = 0.00%), as shown in **Fig 3**. Out of eight studies [21–28] that assessed patients with RA who received upadacitinib, 22 out of 1,743 (1.26%) in the treatment group and 2 out of 782 (0.26%) control subjects developed severe infection [RR 2.05 (95% CI: 0.76–5.55, *I*^*2*^ = 0.00%)].

Six studies [29–31, 33–35] assessed patients with RA who received baricitinib, in which 36 out of 1,953 (1.84%) patients in the treatment group and 26 out of 1,394 (1.87%) control subjects developed severe infection [RR 0.90 (95% CI: 0.54–1.51, *I*^*2*^ = 0.00%)].

Four studies [36–38, 40] assessed patients with RA who received filgotinib, in which 34 out of 2,089 (1.62%) patients in the treatment group and 15 out of 1,111 (1.35%) control subjects developed severe infection [RR 1.19 (95% CI: 0.64–2.19, *I*^*2*^ = 0.00%)].

Out of ten studies [41–50] that assessed tofacitinib in patients with RA, 26 out of 3,114 (0.83%) patients in the treatment group and 3 out of 891 (0.34%) control subjects developed severe infection [RR 1.19 (95% CI: 0.48–2.95, *I*^*2*^ = 0.00%)].

Five studies [51–55] assessed peficitinib in patients with RA, in which 8 out of 1,323 (0.60%) patients in the treatment group and 0 out of 450 (0.00%) control subjects developed severe infection [RR 1.23 (95% CI: 0.29–5.12, *I*^*2*^ = 0.00%)].

#### 3.3.4 Pooled cumulative incidence of severe infection in patients with RA receiving JAKi

The pooled cumulative incidence of severe infection from study initiation until primary outcome assessment, over a mean duration of 17.5 weeks, in 10,730 patients with RA receiving JAKi was 0.92% (95% CI: 0.65%-1.23%, *I*^*2*^ = 43.8%) (**S3 Fig**). The pooled cumulative incidence of severe infection during follow-up, over a mean duration of 30.8 weeks, in 6,835 patients with RA treated with JAKi was 2.02% (95% CI: 1.22%-3.01%, *I*^*2*^ = 84.3%) (**S4 Fig**). The pooled cumulative incidence of severe infection from study initiation until primary outcome assessment, as well as during follow-up, for every JAKi under consideration, is presented in the **S1 File**.

#### 3.3.5 Relative risk and pooled cumulative incidence of opportunistic infections in patients with RA receiving JAKi compared with control subjects

**Fig 4:**
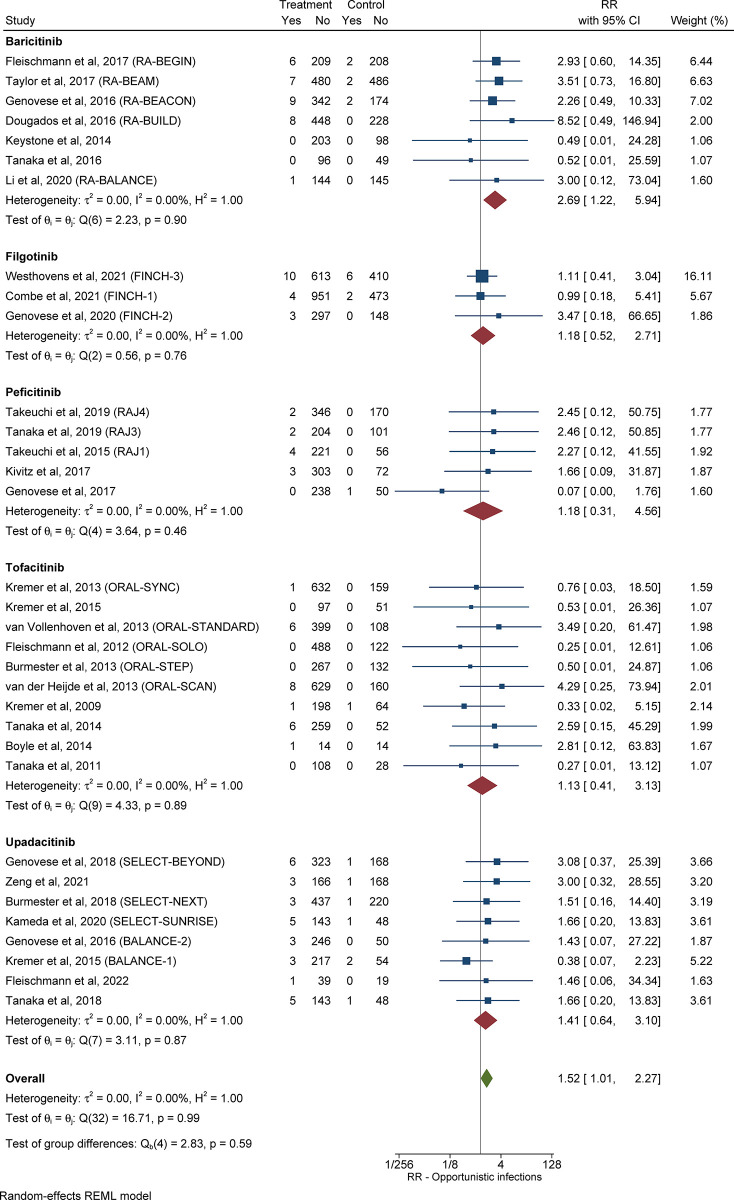
Relative risk of opportunistic infections in patients with RA treated with JAKi compared with control subjects. The size of the square is representative of the weight assigned to the respective study for outcome analysis. The 95% CI for each study is represented by the horizontal lines. The diamond shape represents the pooled estimate of the analysis. Abbreviations: CI: confidence interval; JAKi: Janus-activated kinase inhibitor; RA: rheumatoid arthritis; RR: relative risk.

The RR of opportunistic infections in patients with RA in the treatment group compared control subjects was 1.52 (95% CI: 1.01–2.27, *I*^*2*^ = 0.00%), as shown in **Fig 4**. Information about the pooled cumulative incidence of opportunistic infections in patients with RA treated with JAKi from study initiation until primary outcome assessment is provided in **Table 2** and **S5 Fig**. The pooled cumulative incidence of opportunistic infections during follow-up in 6,835 patients with RA treated with JAKi, over a mean duration of 30.8 weeks, was 1.74% (95% CI: 0.71%-3.16%, *I*^*2*^ = 92.8%) (**S6 Fig**). The RR and pooled cumulative incidence of opportunistic infections from study initiation until primary outcome assessment, as well as during follow-up, for every JAKi under consideration, are presented in the **S1 File**.

#### 3.3.6 Relative risk and pooled cumulative incidence of herpes zoster in patients with RA receiving JAKi compared with control subjects

**Fig 5:**
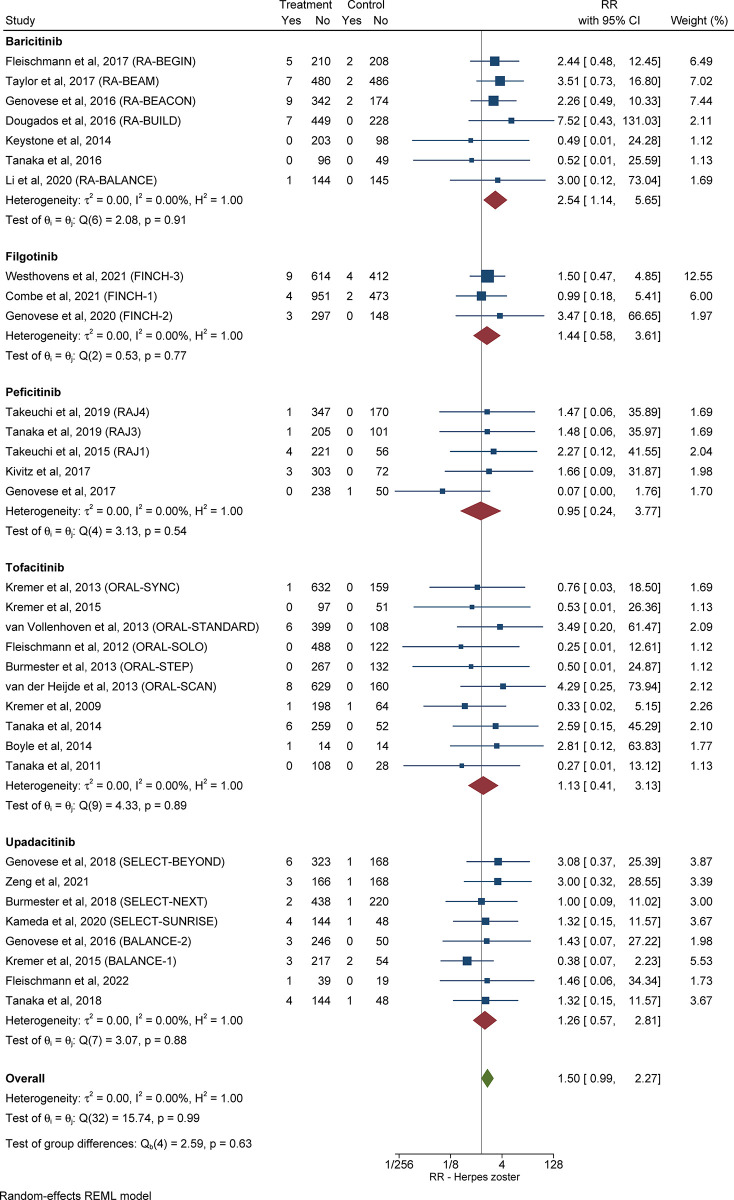
Relative risk of herpes zoster in patients with RA treated JAKi compared with control subjects. The size of the square is representative of the weight assigned to the respective study for outcome analysis. The 95% CI for each study is represented by the horizontal lines. The diamond shape represents the pooled estimate of the analysis. Abbreviations: CI: confidence interval; JAKi: Janus-activated kinase inhibitor; RA: rheumatoid arthritis; RR: relative risk.

The RR of herpes zoster in patients with RA in the treatment group compared with control subjects was 1.50 (95% CI: 0.99–2.27, *I*^*2*^ = 0.00%), as shown in **Fig 5**. Information about the pooled cumulative incidence of herpes zoster in patients with RA treated with JAKi from study initiation up until primary outcome assessment is provided in **Table 2** and **S7 Fig**. The pooled cumulative incidence of herpes zoster during follow-up in 6,835 patients with RA treated with JAKi, over a mean duration of 30.8 weeks, was 1.56% (95% CI: 0.59%-2.92%, *I*^*2*^ = 92.8%) (**S8 Fig**). The RR and pooled cumulative incidence of herpes zoster from study initiation until primary outcome assessment, as well as during follow-up, for every JAKi under consideration, are presented in the **S1 File**.

#### 3.3.7 Relative risk and pooled cumulative incidence of pneumonia in patients with RA receiving JAKi compared with control subjects

**Fig 6:**
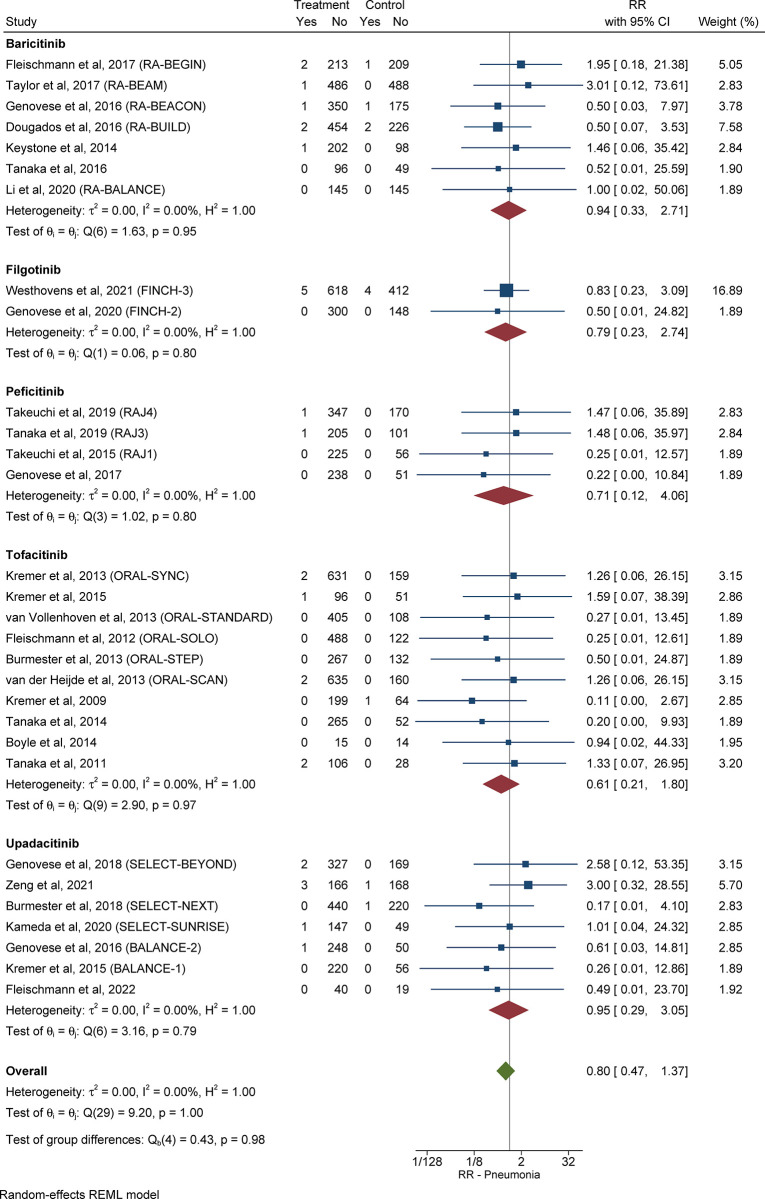
Relative risk of pneumonia in patients with RA treated with JAKi compared with control subjects. The size of the square is representative of the weight assigned to the respective study for outcome analysis. The 95% CI for each study is represented by the horizontal lines. The diamond shape represents the pooled estimate of the analysis. Abbreviations: CI: confidence interval; JAKi: Janus-activated kinase inhibitor; RA: rheumatoid arthritis; RR: relative risk.

The RR of pneumonia in patients with RA in the treatment group compared with control subjects was 0.80 (95% CI: 0.47–1.37, *I*^*2*^ = 0.00%; **Fig 6**). Information about the pooled cumulative incidence of pneumonia in patients with RA treated with JAKi from study initiation up until primary outcome assessment is provided in **Table 2** and **S9 Fig**. The pooled cumulative incidence of pneumonia during follow-up in 6,835 patients with RA treated with JAKi, over a mean duration of 30.8 weeks, was 0.49% (95% CI: 0.20%-0.89%, *I*^*2*^ = 69.1%) (**S10 Fig**). The RR and pooled cumulative incidence of pneumonia from study initiation until primary outcome assessment, as well as during follow-up, for every JAKi under consideration, are presented in the **S1 File**.

### 3.4 Results of meta-regression analysis

When assessing the risk of any-grade, severe, or opportunistic infections in patients with RA treated with JAKi, there was no statistically significant relationship between any type of infection and increasing age, female sex, or concurrent use of glucocorticoids and/or methotrexate (**S4 Table**).

### 3.5 Quality control of studies included in the analysis

Based on the RoB 2 tool, all included randomized controlled trials had a low risk of bias. Detailed assessment of the included studies’ quality metrics is detailed in **S5 Table**. There was no evidence of publication bias in our analysis (bias = 0.46, *p* = 0.4716).

## 4. Discussion

Treatment with JAKi has been shown to slow disease progression and provide symptom relief in patients with RA [56]. However, safety concerns, including a heightened risk of infection, have been reported [57]. In this systematic review and meta-analysis, we found that treatment with baricitinib was associated with an increased risk of any-grade (RR 1.34; 95% CI: 1.19–1.52) and opportunistic (RR 2.69; 95% CI: 1.22–5.94) infection, whereas treatment with filgotinib (RR 1.21; 95% CI: 1.05–1.39), peficitinib (RR 1.40; 95% CI: 1.05–1.86) and upadacitinib (RR 1.30; 95% CI: 1.09–1.56) was associated with increased risk of any-grade infection only.

The JAK-STAT pathway is involved in antiviral and antibacterial host immune responses by modifying type I interferon and interferon-gamma responses as well as effector pathways of major inflammatory interleukins [58]. From our analysis, we found that there was a statistically significant difference in the risk of any-grade but not severe infection in patients with RA in the treatment group receiving baricitinib, filgotinib, peficitinib or upadacitinib, compared to the control group. Consistent with our findings, Bechman *et al*. [59] conducted a meta-analysis of 21 studies to evaluate the incidence of severe infections in patients with RA receiving tofacitinib, baricitinib, or upadacitinib and found that the incidence rate ratio of severe infections in patients receiving JAKi compared to placebo was statistically non-significant. Overall, the incidence rate ratio of severe infections in that study [59] was low (1.97–3.02 per 100 patient-years), which is in line with the cumulative incidence of severe infection in our study, which was approximately 2% during the follow-up period. Similarly, a long-term extension study assessing the safety of tofacitinib over 8.5 years pooled data from 19 clinical trials and reported an incidence rate of severe infections of 2.7 per 100 patient-years [60]. Other long-term extension studies have also reported low long-term severe infection incidence rates [61–63]. Notably, pharmacovigilance studies could provide additional real-world data regarding the infection risk profile of these agents, especially for newly licensed JAKi, such as upadacitinib and filgotinib, which are not extensively studied compared to older JAKi [57].

In our analysis, patients receiving the JAKi baricitinib were significantly more likely to develop opportunistic infections compared to patients in the control group. The increased risk of opportunistic infections in patients receiving JAKi has been attributed to down-modulation of cytokines responsible for mounting T-cell specific responses against pathogens [64], impaired granuloma formation necessary for containment of infections, such as tuberculosis [65], and diminished interferon-mediated antiviral responses that protect against viral infections [66]. The most common infections as a result of JAKi therapy reported in our analysis and other published studies, in addition to herpes zoster, include *Candida* spp., followed by infections from cytomegalovirus, *Cryptococcus* spp., *Pneumocystis jirovecii*, and tuberculosis [57, 67]. The pooled cumulative incidence of these opportunistic infections in our study was below 2%. Winthrop *et al*. [68] pooled the results of 12 clinical trials and two long-term extension studies including 5,671 patients treated with tofacitinib and reported 60 opportunistic infections, translating to an incidence rate of 0.46 per 100 patient-years. Other long-term extension studies have reported a low incidence of opportunistic infections as well [62, 69], reinforcing the favorable long-term infection risk profile of JAKi.

In our analysis, patients treated with any JAKi had a higher risk of herpes zoster compared to controls, but the difference in risk was not statistically significant, except for baricitinib. A previous meta-analysis conducted by Wang *et al*. [70] assessed the safety of JAKi and found that treatment with any JAKi increased herpes zoster risk, whereas a subgroup analysis according to specific JAKi revealed that only baricitinib was associated with a heightened herpes zoster risk. Failure to reveal a significantly increased risk of infection with JAKi other than baricitinib in our and previous studies might be due to confounding variables that are not accounted for, such as area of residence, race/ethnicity, prior history of herpes zoster and vaccination against herpes zoster. Of note, increasing age and concomitant glucocorticoid use, which have been associated with heightened herpes zoster risk [71], were included in our meta-regression analysis and did not affect the association between JAKi use and risk of herpes zoster. Also, during the follow-up period of the included studies, the pooled cumulative incidence of herpes zoster was 1.56%, while the cumulative incidence from study initiation until primary outcome assessment was 0.74%. Due to the lack of a true comparator group during follow-up, the failure to include herpes zoster cases that occurred during the follow-up period in our comparative risk analysis between intervention and control groups might account for the lack of significant findings. Nevertheless, since herpes zoster was the most frequent opportunistic infection in our analysis and has been clearly established as a complication of JAKi therapy, both in terms of pathophysiology [72] and based on previous study experiences [73], it is imperative for clinicians to remain vigilant for this complication.

Our study has certain limitations. First, there was noticeable cross-over of patients in the treatment and control groups after primary outcome assessment in each included study, so we were unable to estimate the long-term infection risk profile of JAKi. Next, due to insufficient data, we could not estimate whether concomitant medications, other than methotrexate and glucocorticoids, as well as prior use of biologic DMARDs or JAKi, could confound the association between current JAKi use and infection risk. Furthermore, the number of patients receiving antibiotic prophylaxis was not available in the included studies, and, as such, the impact of antibiotics on the risk and frequency of infections cannot be evaluated. Also, the diagnostic methods used to identify infections may have been different in the included studies, resulting in variability in the detection rate of infections, including opportunistic infections such as *Pneumocystis jirovecii* [74], *Cryptococcus* spp. [75] and tuberculosis [76]. Finally, information on race/ethnicity was available for the entire population entering each individual study, and no information was provided for participants who developed infections. As such, although geographic residence and race/ethnicity have been associated with varying risk of opportunistic infections, including herpes zoster [71], we could not estimate their impact on infection risk in our analysis.

In summary, patients with RA treated with baricitinib, filgotinib, peficitinib or upadacitinib were at increased risk of any-grade, but not severe, infection compared to patients in the control group. Risk of herpes zoster was increased in the intervention compared to the control group, but the difference in risk was not statistically significant, likely due to the lack of data on long-term follow-up. Well-designed prospective studies and registries should evaluate the effect of vaccination and prophylactic antibiotic regimens on the incidence and outcomes of infection in patients with RA receiving JAKi. Also, as our work focused on elucidating the infection risk profile of JAKi in patients with RA, we did not include information on other aspects of JAKi, including treatment efficacy and non-infection risk profile. In the future, research could focus on covering different aspects of this drug class, including treatment efficacy, safety profile beyond infections, and identification on immune markers that could predict treatment response.

## Supporting information

S1 File Supplementary appendix. (DOCX)

S2 File PRISMA checklist. (DOCX)

S1 Fig Pooled cumulative incidence of any-grade infection in patients with RA treated with JAKi, from study initiation until primary study outcome assessment, with 95% CI. The red dotted line represents the overall pooled estimate of the cumulative incidence of any-grade infection in patients treated with JAKi from study initiation until primary study outcome assessment, while the weight percentages correspond to the contribution of each study to the pooled estimate. Abbreviations: CI: confidence interval; ES: effect size; JAKi: Janus-activated kinase inhibitor; RA: rheumatoid arthritis.
(TIF)

S2 Fig Pooled cumulative incidence of any-grade infection in patients with RA treated with JAKi, during follow-up extending from the time of primary study outcome assessment until the end of the study, with 95% CI. The red dotted line represents the overall pooled estimate of the cumulative incidence of any-grade infection in patients treated with JAKi, during follow-up extending from the time of primary study outcome assessment until the end of the study, while the weight percentages correspond to the contribution of each study to the pooled estimate. Abbreviations: CI: confidence interval; ES: effect size; JAKi: Janus-activated kinase inhibitor; RA: rheumatoid arthritis.
(TIF)

S3 Fig Pooled cumulative incidence of severe infection in patients with RA treated with JAKi, from study initiation until primary study outcome assessment, with 95% CI. The red dotted line represents the overall pooled estimate of the cumulative incidence of severe infection in patients treated with JAKi from study initiation until primary study outcome assessment, while the weight percentages correspond to the contribution of each study to the pooled estimate. Abbreviations: CI: confidence interval; ES: effect size; JAKi: Janus-activated kinase inhibitor; RA: rheumatoid arthritis.
(TIF)

S4 Fig Pooled cumulative incidence of severe infection in patients with RA treated with JAKi, during follow-up extending from the time of primary study outcome assessment until the end of the study, with 95% CI. The red dotted line represents the overall pooled estimate of the cumulative incidence of severe infection in patients treated with JAKi, during follow-up extending from the time of primary study outcome assessment until the end of the study, while the weight percentages correspond to the contribution of each study to the pooled estimate. Abbreviations: CI: confidence interval; ES: effect size; JAKi: Janus-activated kinase inhibitor; RA: rheumatoid arthritis.
(TIF)

S5 Fig Pooled cumulative incidence of opportunistic infections in patients with RA treated with JAKi, from study initiation until primary study outcome assessment, with 95% CI. The red dotted line represents the overall pooled estimate of the cumulative incidence of opportunistic infections infection in patients treated with JAKi from study initiation until primary study outcome assessment, while the weight percentages correspond to the contribution of each study to the pooled estimate. Abbreviations: CI: confidence interval; ES: effect size; JAKi: Janus-activated kinase inhibitor; RA: rheumatoid arthritis.
(TIF)

S6 Fig Pooled cumulative incidence of opportunistic infections in patients with RA treated with JAKi, during follow-up extending from the time of primary study outcome assessment until the end of the study, with 95% CI. The red dotted line represents the overall pooled estimate of the cumulative incidence of opportunistic infections infection in patients treated with JAKi, during follow-up extending from the time of primary study outcome assessment until the end of the study, while the weight percentages correspond to the contribution of each study to the pooled estimate. Abbreviations: CI: confidence interval; ES: effect size; JAKi: Janus-activated kinase inhibitor; RA: rheumatoid arthritis.
(TIF)

S7 Fig Pooled cumulative incidence of herpes zoster in patients with RA treated with JAKi, from study initiation until primary study outcome assessment, with 95% CI. The red dotted line represents the overall pooled estimate of the cumulative incidence of herpes zoster in patients treated with JAKi from study initiation until primary study outcome assessment, while the weight percentages correspond to the contribution of each study to the pooled estimate. Abbreviations: CI: confidence interval; ES: effect size; JAKi: Janus-activated kinase inhibitor; RA: rheumatoid arthritis.
(TIF)

S8 Fig Pooled cumulative incidence of herpes zoster in patients with RA treated with JAKi, during follow-up extending from the time of primary study outcome assessment until the end of the study, with 95% CI. The red dotted line represents the overall pooled estimate of the cumulative incidence of herpes in patients treated with JAKi, during follow-up extending from the time of primary study outcome assessment until the end of the study, while the weight percentages correspond to the contribution of each study to the pooled estimate. Abbreviations: CI: confidence interval; ES: effect size; JAKi: Janus-activated kinase inhibitor; RA: rheumatoid arthritis.
(TIF)

S9 Fig Pooled cumulative incidence of pneumonia in patients with RA treated with JAKi, from study initiation until primary study outcome assessment, with 95% CI. The red dotted line represents the overall pooled estimate of the cumulative incidence of pneumonia in patients treated with JAKi from study initiation until primary study outcome assessment, while the weight percentages correspond to the contribution of each study to the pooled estimate. Abbreviations: CI: confidence interval; ES: effect size; JAKi: Janus-activated kinase inhibitor; RA: rheumatoid arthritis.
(TIF)

S10 Fig Pooled cumulative incidence of pneumonia in patients with RA treated with JAKi, during follow-up extending from the time of primary study outcome assessment until the end of the study, with 95% CI. The red dotted line represents the overall pooled estimate of the cumulative incidence of pneumonia in patients treated with JAKi, during follow-up extending from the time of primary study outcome assessment until the end of the study, while the weight percentages correspond to the contribution of each study to the pooled estimate. Abbreviations: CI: confidence interval; ES: effect size; JAKi: Janus-activated kinase inhibitor; RA: rheumatoid arthritis.
(TIF)

S1 Table Characteristics of the randomized controlled trials included in the analysis. (PDF)

S2 Table Number and type of infections in patients with RA treated with JAKi from study initiation until primary study outcome assessment compared to patients in the control group. (PDF)

S3 Table Number and type of infections in patients with RA treated with JAKi during follow-up, extending from the time of primary study outcome assessment until the end of the study compared to patients in the control group. (PDF)

S4 Table Random-effects meta-regression analysis for age, sex, current use of MTX and/or corticosteroids and risk of any-grade, severe, or opportunistic infection in patients with RA treated with JAKi compared to patients in the control group. (PDF)

S5 Table Quality assessment of the included studies using the RoB 2 tool. (PDF)
