# Supplementary material for: Cumulative incidence and risk of infection in patients with rheumatoid arthritis treated with janus kinase inhibitors: A systematic review and meta-analysis
Source: PLoS One. 2024 Jul 31;19(7):e0306548. doi: 10.1371/journal.pone.0306548 (PMC11290652; doi:10.1371/journal.pone.0306548)
Supplement: S1 File — (DOCX) [file pone.0306548.s001.docx]

***PLOS One***

**Supplementary Appendix**

**Supplement to:** Cumulative Incidence and Risk of Infection in Patients with Rheumatoid Arthritis Treated with Janus-activated Kinase Inhibitors: A Systematic Review and Meta-analysis

Konstantinos Ouranos^1^, Diana V Avila^1^, Evangelia K Mylona^1^, Athanasios Vassilopoulos^2^, Stephanos Vassilopoulos^2^, Fadi Shehadeh^1,3^, Eleftherios Mylonakis^1,4^

^1^Department of Medicine, Houston Methodist Research Institute, Houston, TX, USA

^2^Department of Medicine, Warren Alpert Medical School of Brown University, Rhode Island Hospital, Providence, RI, USA

^3^School of Electrical and Computer Engineering, National Technical University of Athens, Athens, Greece

^4^Weill Cornell Medicine, New York, NY, USA.

**Correspondence**: Eleftherios Mylonakis, M.D., Ph.D., FIDSA; Chair, Department of Medicine, Houston Methodist Hospital, 6550 Fannin Street, Smith Tower 1001, Houston, Texas 77030 ([emylonakis@houstonmethodist.org](mailto:emylonakis@houstonmethodist.org))

**Contents**

**Supplementary Results**

**Pooled cumulative incidence of any-grade infection in patients with RA receiving JAKi**

The pooled cumulative incidence of any-grade infection in 1,743 patients with RA receiving upadacitinib from study initiation until primary outcome assessment (mean duration: 12 weeks) was 20.71% (95% CI: 14.40%-27.80%, *I^2^* = 91.20%), whereas the pooled cumulative incidence during follow-up in 515 patients (mean duration: 50 weeks) was 67.74% (95% CI: 63.63%-71.72%). The pooled cumulative incidence of any-grade infection in 1,953 with RA treated with baricitinib until primary outcome assessment (mean duration: 24.6 weeks) was 33.40% (95% CI: 24.40%-40.95%, *I^2^* = 93.30%), whereas the pooled cumulative incidence during follow-up in 1,341 patients with RA (mean duration: 27 weeks) was 38.33% (95% CI: 9.46%-72.76%, *I^2^* = 99.3%). The pooled cumulative incidence of any-grade infection in 2,386 patients with RA receiving filgotinib until primary outcome assessment (mean duration: 31 weeks) was 21.91% (95% CI: 14.97%-29.76%, *I^2^* = 94.70%), whereas the pooled cumulative incidence during follow-up in 1,181 patients (mean duration: 20 weeks) was 38.08% (95% CI: 35.32%-40.87%,). The pooled cumulative incidence of any-grade infection in 3,114 patients with RA treated with tofacitinib until primary outcome assessment (mean duration: 11.6 weeks) was 14.40% (95% CI: 11.67%-17.36%, *I^2^* = 76.90%), whereas the pooled cumulative incidence of any-grade infection during follow-up in 2,871 patients with RA (mean duration: 16.8 weeks) was 13.15% (95% CI: 9.30%-17.56%, *I^2^* = 90.9%). Finally, the pooled cumulative incidence of any-grade infection in 779 patients with RA treated with peficitinib until primary outcome assessment (mean duration: 12 weeks) was 22.38% (95% CI: 19.22%-25.71%), whereas the pooled cumulative incidence of any-grade infection during follow-up (mean duration: 40 weeks) in 927 patients was 37.27% (95% CI: 34.18%-40.42%).

**Pooled cumulative incidence of severe infection in patients with RA receiving JAKi**

The pooled cumulative incidence of severe infection in 1,743 patients with RA receiving upadacitinib from study initiation until primary outcome assessment (mean duration: 12 weeks) was 0.99% (95% CI: 0.34%-1.90%, *I^2^* = 49.5%), whereas the pooled cumulative incidence during follow-up in 515 patients (mean duration: 50 weeks) was 7.85% (95% CI: 5.66%-10.36%). The pooled cumulative incidence of severe infection in 1,953 with RA treated with baricitinib until primary outcome assessment (mean duration: 24.6 weeks) was 1.66% (95% CI: 1.00%-2.45%, *I^2^* = 26.3%), whereas the pooled cumulative incidence during follow-up in 1,341 patients with RA (mean duration: 27 weeks) was 1.95% (95% CI: 0.76%-3.61%, *I^2^* = 63.5%). The pooled cumulative incidence of severe infection in 2,597 patients with RA receiving filgotinib until primary outcome assessment (mean duration: 27.2 weeks) was 1.54% (95% CI: 1.09%-2.07% *I^2^* = 0.00%), whereas the pooled cumulative incidence during follow-up in 1,181 patients (mean duration: 20 weeks) was 2.58% (95% CI: 1.73%-3.58%). The pooled cumulative incidence of severe infection in 3,114 patients with RA treated with tofacitinib until primary outcome assessment (mean duration: 11.6 weeks) was 0.41% (95% CI: 0.08%-0.92%, *I^2^* = 44.40%), whereas the pooled cumulative incidence of severe infection during follow-up in 2,871 patients with RA (mean duration: 16.8 weeks) was 1.05% (95% CI: 0.48%-1.82%, *I^2^* = 66.2%). Finally, the pooled cumulative incidence of severe infection in 1,323 patients with RA treated with peficitinib until primary outcome assessment (mean duration: 12 weeks) was 0.52% (95% CI: 0.15%-1.03%, *I^2^* = 0.00%), whereas the pooled cumulative incidence of severe infection during follow-up (mean duration: 40 weeks) in 927 patients was 1.27% (95% CI: 0.62%-2.13%).

**Relative risk and pooled cumulative incidence of opportunistic infections in patients with RA receiving JAKi**

Among eight studies [1-8] that evaluated patients with RA who received upadacitinib, 29 out of 1,743 (1.66%) patients in the treatment group and 7 out of 782 (0.90%) patients in the control group developed opportunistic infections, with RR of 1.41 (95% CI: 0.64-3.10, *I^2^* = 0.00%). The pooled cumulative incidence of opportunistic infections in 515 patients with RA receiving upadacitinib (mean duration: 50 weeks) was 9.63% (95% CI: 7.21%-12.35%). For seven studies [9-15] that evaluated patients with RA who received baricitinib, 31 out of 1,953 (1.59%) patients in the treatment group and 6 out of 1,394 (0.43%) patients in the control group developed opportunistic infections, with RR of 2.69 (95% CI: 1.22-5.94, *I^2^* = 0.00%). The pooled cumulative incidence of opportunistic infections in 1,341 patients with RA receiving baricitinib during follow-up (mean duration: 27 weeks) was 1.10% (95% CI: 0.00%-4.48%, *I^2^* = 91.9%). Among three [16-18] studies that evaluated patients with RA who received filgotinib, 17 out of 1,878 (0.91%) patients in the treatment group and 8 out of 1,039 (0.77%) patients in the control group developed opportunistic infections, with RR of 1.18 (95% CI: 0.52-2.71, *I^2^* = 0.00%). The pooled cumulative incidence of opportunistic infections in 1,181 patients with RA receiving filgotinib during follow-up (mean duration: 20 weeks) was 0.87% (95% CI: 0.39%-1.52%). Next, among ten studies [19-28] that assessed tofacitinib in patients with RA, 23 out of 3,114 (0.74%) of patients in the treatment group and 1 out of 891 (0.11%) patients in the control group developed opportunistic infections, with RR of 1.13 (95% CI: 0.41-3.13, *I^2^* = 0.00%). The pooled cumulative incidence of opportunistic infections in 2,871 patients with RA receiving tofacitinib (mean duration: 16.8 weeks) was 0.56% (95% CI: 0.04%-1.54%, *I^2^* = 85.0%). Finally, among five studies [29-33] that evaluated peficitinib in patients with RA, 11 out of 1,323 (0.83%) patients in the treatment group and 1 out of 450 (0.22%) patients in the control group developed opportunistic infections, with RR of 1.18 (95% CI: 0.31-4.56, *I^2^* = 0.00%). The pooled cumulative incidence of opportunistic infections in 927 patients with RA receiving peficitinib (mean duration: 40 weeks) was 3.39% (95% CI: 2.30%-4.67%).

**Relative risk and pooled cumulative incidence of herpes zoster in patients with RA receiving JAKi**

Among eight studies [1-8] that evaluated patients with RA who received upadacitinib, 29 out of 1,743 (1.66%) patients in the treatment group and 7 out of 782 (0.90%) patients in the control group developed herpes zoster, with RR of 1.26 (95% CI: 0.57-2.81, *I^2^* = 0.00%). The pooled cumulative incidence of herpes zoster in 515 patients with RA receiving upadacitinib (mean duration: 50 weeks) was 8.89% (95% CI: 6.56%-11.53%). For seven studies [9-15] that evaluated patients with RA who received baricitinib, 29 out of 1,953 (1.48%) patients in the treatment group and 6 out of 1,394 (0.31%) patients in the control group developed herpes zoster, with RR of 2.54 (95% CI: 1.14-5.65, *I^2^* = 0.00%). The pooled cumulative incidence of herpes zoster in 1,341 patients with RA receiving baricitinib during follow-up (mean duration: 27 weeks) was 1.10% (95% CI: 0.00%-4.16%, *I^2^* = 90.6%). Among three [16-18] studies that evaluated patients with RA who received filgotinib, 17 out of 1,878 (0.91%) patients in the treatment group and 8 out of 1,039 (0.77%) patients in the control group developed herpes zoster, with RR of 1.44 (95% CI: 0.58-3.61, *I^2^* = 0.00%). The pooled cumulative incidence of opportunistic infections in 1,181 patients with RA receiving filgotinib during follow-up (mean duration: 20 weeks) was 0.87% (95% CI: 0.39%-1.52%). Next, among ten studies [19-28] that assessed tofacitinib in patients with RA, 23 out of 3,114 (0.74%) of patients in the treatment group and 0 out of 891 (0%) patients in the control group developed herpes zoster, with RR of 1.13 (95% CI: 0.41-3.13, *I^2^* = 0.00%). The pooled cumulative incidence of opportunistic infections in 2,871 patients with RA receiving tofacitinib (mean duration: 16.8 weeks) was 0.35% (95% CI: 0.00%-1.35%, *I^2^* = 88.3%). Finally, among five studies [29-33] that evaluated peficitinib in patients with RA, 9 out of 1,323 (0.68%) patients in the treatment group and 1 out of 450 (0.22%) patients in the control group developed herpes zoster, with RR of 0.95 (95% CI: 0.24-3.77, *I^2^* = 0.00%). The pooled cumulative incidence of herpes zoster in 927 patients with RA receiving peficitinib (mean duration: 40 weeks) was 3.27% (95% CI: 2.20%-4.54%).

**Relative risk and pooled cumulative incidence of pneumonia in patients with RA receiving JAKi**

Among seven studies [1-7] that evaluated patients with RA who received upadacitinib, 7 out of 1,595 (0.44%) patients in the treatment group and 2 out of 733 (0.27%) patients in the control group developed pneumonia, with RR of 0.95 (95% CI: 0.29-3.05, *I^2^* = 0.00%). The pooled cumulative incidence of pneumonia in 515 patients with RA receiving upadacitinib (mean duration: 50 weeks) was 2.37% (95% CI: 1.18%-3.91%). For seven studies [9-15] that evaluated patients with RA who received baricitinib, 7 out of 1,953 (0.36%) patients in the treatment group and 4 out of 1,394 (0.29%) patients in the control group developed pneumonia, with RR of 0.94 (95% CI: 0.33-2.71, *I^2^* = 0.00%). The pooled cumulative incidence of pneumonia in 1,341 patients with RA receiving baricitinib during follow-up (mean duration: 27 weeks) was 0.48% (95% CI: 0.05%-1.21%, *I^2^* = 37.0%). Among two studies [17, 18] that evaluated patients with RA who received filgotinib, 5 out of 923 (0.54%) patients in the treatment group and 4 out of 564 (0.71%) patients in the control group developed pneumonia, with RR of 0.79 (95% CI: 0.23-2.74, *I^2^* = 0.00%). The pooled cumulative incidence of pneumonia in 1,181 patients with RA receiving filgotinib during follow-up (mean duration: 20 weeks) was 0.70% (95% CI: 0.27%-1.30%). Next, among ten studies [19-28] that assessed tofacitinib in patients with RA, 7 out of 3,114 (0.22%) of patients in the treatment group and 1 out of 891 (0.11%) patients in the control group developed pneumonia, with RR of 0.61 (95% CI: 0.21-1.80, *I^2^* = 0.00%). The pooled cumulative incidence of opportunistic infections in 2,871 patients with RA receiving tofacitinib (mean duration: 16.8 weeks) was 0.12% (95% CI: 0.00%-0.34%, *I^2^* = 23.7%). Finally, among four studies [30-33] that evaluated peficitinib in patients with RA, 2 out of 1,017 (0.20%) patients in the treatment group and 0 out of 378 (0%) patients in the control group developed pneumonia, with RR of 0.71 (95% CI: 0.12-4.06, *I^2^* = 0.00%). The pooled cumulative incidence of pneumonia in 927 patients with RA receiving peficitinib (mean duration: 40 weeks) was 0.74% (95% CI: 0.25%-1.43%).

**References**

1. Ramsey, A., C. Rozario, and J. Stern, *Direct challenges are the gold standard for most antibiotic allergy evaluations.* Ann Allergy Asthma Immunol, 2023. **131**(4): p. 427-433.

2. Burmester, G.R., et al., *Safety and efficacy of upadacitinib in patients with rheumatoid arthritis and inadequate response to conventional synthetic disease-modifying anti-rheumatic drugs (SELECT-NEXT): a randomised, double-blind, placebo-controlled phase 3 trial.* Lancet, 2018. **391**(10139): p. 2503-2512.

3. H, K., et al., *Efficacy and safety of upadacitinib in Japanese patients with rheumatoid arthritis (SELECT-SUNRISE): a placebo-controlled phase IIb/III study*. Vol. 59. 2020, England. 3303-3313.

4. Kremer, J.M., et al., *A Phase IIb Study of ABT-494, a Selective JAK-1 Inhibitor, in Patients With Rheumatoid Arthritis and an Inadequate Response to Anti-Tumor Necrosis Factor Therapy.* Arthritis Rheumatol, 2016. **68**(12): p. 2867-2877.

5. MC, G., et al., *Efficacy and Safety of ABT-494, a Selective JAK-1 Inhibitor, in a Phase IIb Study in Patients With Rheumatoid Arthritis and an Inadequate Response to Methotrexate*. Vol. 68. 2016, United States. 2857-2866.

6. MC, G., et al., *Safety and efficacy of upadacitinib in patients with active rheumatoid arthritis refractory to biologic disease-modifying anti-rheumatic drugs (SELECT-BEYOND): a double-blind, randomised controlled phase 3 trial*. Vol. 391. 2018, England. 2513-2524.

7. X, Z., et al., *Upadacitinib in patients from China, Brazil, and South Korea with rheumatoid arthritis and an inadequate response to conventional therapy.* 2021. **24**(12): p. 1530-1539.

8. Tanaka, Y., et al., *SAT0257 A phase 2b/3 randomised, placebo-controlled, double-blind study of upadacitinib, a selective jak1 inhibitor, in japanese patients with active rheumatoid arthritis and inadequate response to conventional synthetic dmards.* 2018. **77**(Suppl 2): p. 991-992.

9. Dougados, M., et al., *Baricitinib in patients with inadequate response or intolerance to conventional synthetic DMARDs: results from the RA-BUILD study.* Ann Rheum Dis, 2017. **76**(1): p. 88-95.

10. Li, Z., et al., *Baricitinib in patients with rheumatoid arthritis with inadequate response to methotrexate: results from a phase 3 study.* Clin Exp Rheumatol, 2020. **38**(4): p. 732-741.

11. Keystone, E.C., et al., *Safety and efficacy of baricitinib at 24 weeks in patients with rheumatoid arthritis who have had an inadequate response to methotrexate.* Ann Rheum Dis, 2015. **74**(2): p. 333-40.

12. Fleischmann, R., et al., *Baricitinib, Methotrexate, or Combination in Patients With Rheumatoid Arthritis and No or Limited Prior Disease-Modifying Antirheumatic Drug Treatment.* Arthritis Rheumatol, 2017. **69**(3): p. 506-517.

13. MC, G., et al., *Baricitinib in Patients with Refractory Rheumatoid Arthritis*. Vol. 374. 2016, United States. 1243-52.

14. Taylor, P.C., et al., *Baricitinib versus Placebo or Adalimumab in Rheumatoid Arthritis.* N Engl J Med, 2017. **376**(7): p. 652-662.

15. Y, T., et al., *Efficacy and Safety of Baricitinib in Japanese Patients with Active Rheumatoid Arthritis Receiving Background Methotrexate Therapy: A 12-week, Double-blind, Randomized Placebo-controlled Study*. Vol. 43. 2016, Canada. 504-11.

16. Combe, B., et al., *Filgotinib versus placebo or adalimumab in patients with rheumatoid arthritis and inadequate response to methotrexate: a phase III randomised clinical trial.* Ann Rheum Dis, 2021. **80**(7): p. 848-858.

17. MC, G., et al., *Effect of Filgotinib vs Placebo on Clinical Response in Patients With Moderate to Severe Rheumatoid Arthritis Refractory to Disease-Modifying Antirheumatic Drug Therapy: The FINCH 2 Randomized Clinical Trial*. Vol. 322. 2019, United States. 315-325.

18. R, W., et al., *Filgotinib in combination with methotrexate or as monotherapy versus methotrexate monotherapy in patients with active rheumatoid arthritis and limited or no prior exposure to methotrexate: the phase 3, randomised controlled FINCH 3 trial*. Vol. 80. 2021, England. 727-738.

19. Boyle, D.L., et al., *The JAK inhibitor tofacitinib suppresses synovial JAK1-STAT signalling in rheumatoid arthritis.* Ann Rheum Dis, 2015. **74**(6): p. 1311-6.

20. Burmester, G.R., et al., *Tofacitinib (CP-690,550) in combination with methotrexate in patients with active rheumatoid arthritis with an inadequate response to tumour necrosis factor inhibitors: a randomised phase 3 trial.* Lancet, 2013. **381**(9865): p. 451-60.

21. D, v.d.H., et al., *Tofacitinib (CP-690,550) in patients with rheumatoid arthritis receiving methotrexate: twelve-month data from a twenty-four-month phase III randomized radiographic study*. Vol. 65. 2013, United States. 559-70.

22. JM, K., et al., *Evaluation of the effect of tofacitinib on measured glomerular filtration rate in patients with active rheumatoid arthritis: results from a randomised controlled trial*. Vol. 17. 2015, England. 95.

23. Kremer, J., et al., *Tofacitinib in combination with nonbiologic disease-modifying antirheumatic drugs in patients with active rheumatoid arthritis: a randomized trial.* Ann Intern Med, 2013. **159**(4): p. 253-61.

24. Kremer, J.M., et al., *The safety and efficacy of a JAK inhibitor in patients with active rheumatoid arthritis: Results of a double-blind, placebo-controlled phase IIa trial of three dosage levels of CP-690,550 versus placebo.* Arthritis Rheum, 2009. **60**(7): p. 1895-905.

25. Strand, V., et al., *SAT0256 Tofacitinib with and without methotrexate versus adalimumab with methotrexate for the treatment of rheumatoid arthritis: patient-reported outcomes from a phase 3b/4 randomised trial*, in *Saturday, 16 JUNE 2018*. 2018. p. 990.2-991.

26. Y, T., et al., *Phase II study of tofacitinib (CP-690,550) combined with methotrexate in patients with rheumatoid arthritis and an inadequate response to methotrexate*. Vol. 63. 2011, United States. 1150-8.

27. Y, T., et al., *Efficacy and safety of tofacitinib as monotherapy in Japanese patients with active rheumatoid arthritis: a 12-week, randomized, phase 2 study*. Vol. 25. 2015, England. 514-21.

28. van Vollenhoven, R.F., et al., *Tofacitinib or adalimumab versus placebo in rheumatoid arthritis.* N Engl J Med, 2012. **367**(6): p. 508-19.

29. AJ, K., et al., *Peficitinib, a JAK Inhibitor, in the Treatment of Moderate-to-Severe Rheumatoid Arthritis in Patients With an Inadequate Response to Methotrexate*. Vol. 69. 2017, United States. 709-719.

30. Genovese, M.C., et al., *Peficitinib, a JAK Inhibitor, in Combination With Limited Conventional Synthetic Disease-Modifying Antirheumatic Drugs in the Treatment of Moderate-to-Severe Rheumatoid Arthritis.* Arthritis Rheumatol, 2017. **69**(5): p. 932-942.

31. T, T., et al., *Efficacy and safety of the oral Janus kinase inhibitor peficitinib (ASP015K) monotherapy in patients with moderate to severe rheumatoid arthritis in Japan: a 12-week, randomised, double-blind, placebo-controlled phase IIb study*. Vol. 75. 2016, England. 1057-64.

32. T, T., et al., *Efficacy and safety of peficitinib (ASP015K) in patients with rheumatoid arthritis and an inadequate response to methotrexate: results of a phase III randomised, double-blind, placebo-controlled trial (RAJ4) in Japan*. Vol. 78. 2019, England. 1305-1319.

33. Tanaka, Y., et al., *Efficacy and safety of peficitinib (ASP015K) in patients with rheumatoid arthritis and an inadequate response to conventional DMARDs: a randomised, double-blind, placebo-controlled phase III trial (RAJ3).* Ann Rheum Dis, 2019. **78**(10): p. 1320-1332.
