# Supplementary figures and images for: Cumulative incidence and risk of infection in patients with rheumatoid arthritis treated with janus kinase inhibitors: A systematic review and meta-analysis
Source: PLoS One. 2024 Jul 31;19(7):e0306548. doi: 10.1371/journal.pone.0306548 (PMC11290652; doi:10.1371/journal.pone.0306548)

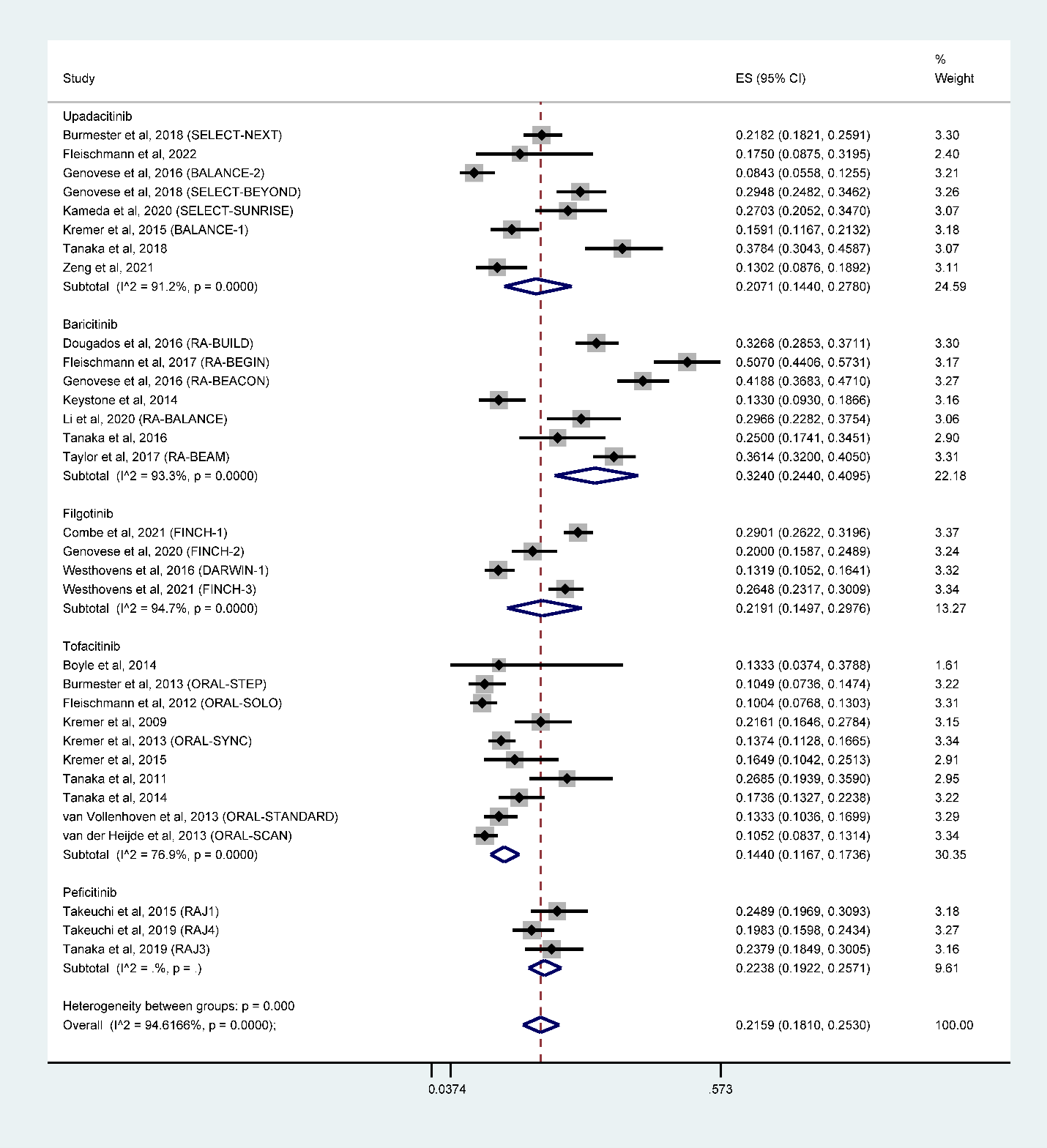

Supplement: S1 Fig — The red dotted line represents the overall pooled estimate of the cumulative incidence of any-grade infection in patients treated with JAKi from study initiation until primary study outcome assessment, while the weight percentages correspond to the contribution of each study to the pooled estimate. Abbreviations: CI: confidence interval; ES: effect size; JAKi: Janus-activated kinase inhibitor; RA: rheumatoid arthritis. (TIF) [file pone.0306548.s003.tif]

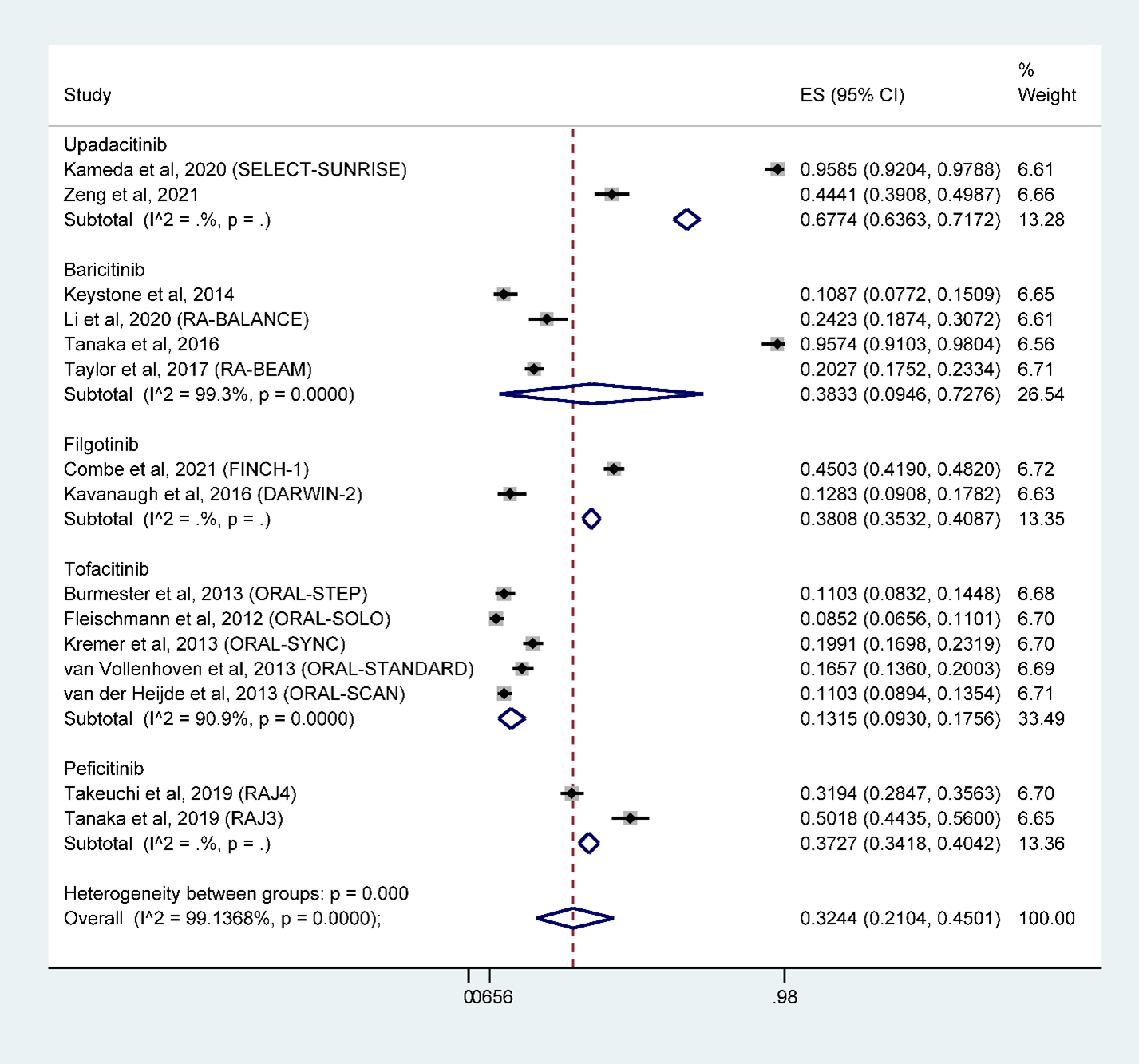

Supplement: S2 Fig — The red dotted line represents the overall pooled estimate of the cumulative incidence of any-grade infection in patients treated with JAKi, during follow-up extending from the time of primary study outcome assessment until the end of the study, while the weight percentages correspond to the contribution of each study to the pooled estimate. Abbreviations: CI: confidence interval; ES: effect size; JAKi: Janus-activated kinase inhibitor; RA: rheumatoid arthritis. (TIF) [file pone.0306548.s004.tif]

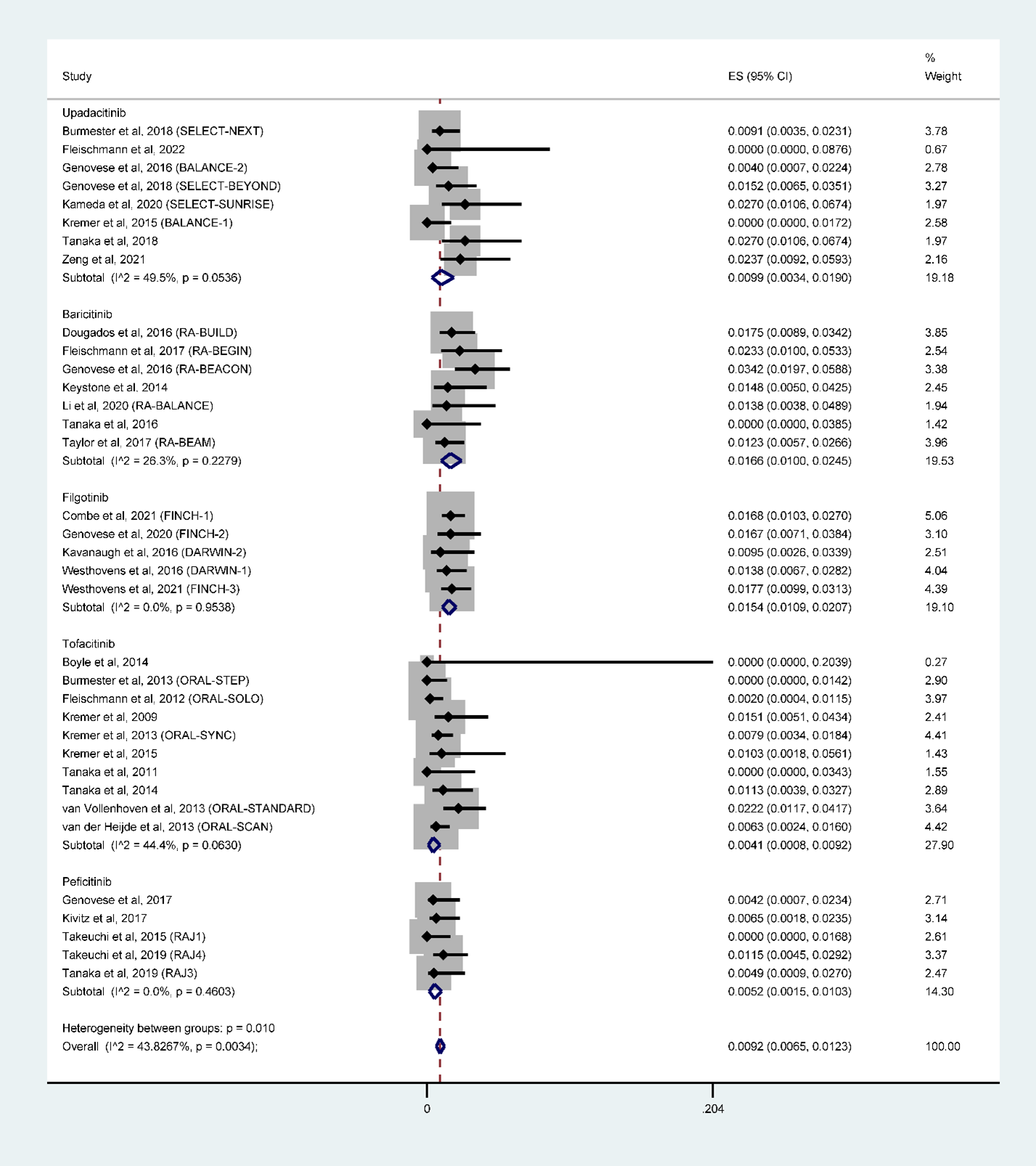

Supplement: S3 Fig — The red dotted line represents the overall pooled estimate of the cumulative incidence of severe infection in patients treated with JAKi from study initiation until primary study outcome assessment, while the weight percentages correspond to the contribution of each study to the pooled estimate. Abbreviations: CI: confidence interval; ES: effect size; JAKi: Janus-activated kinase inhibitor; RA: rheumatoid arthritis. (TIF) [file pone.0306548.s005.tif]

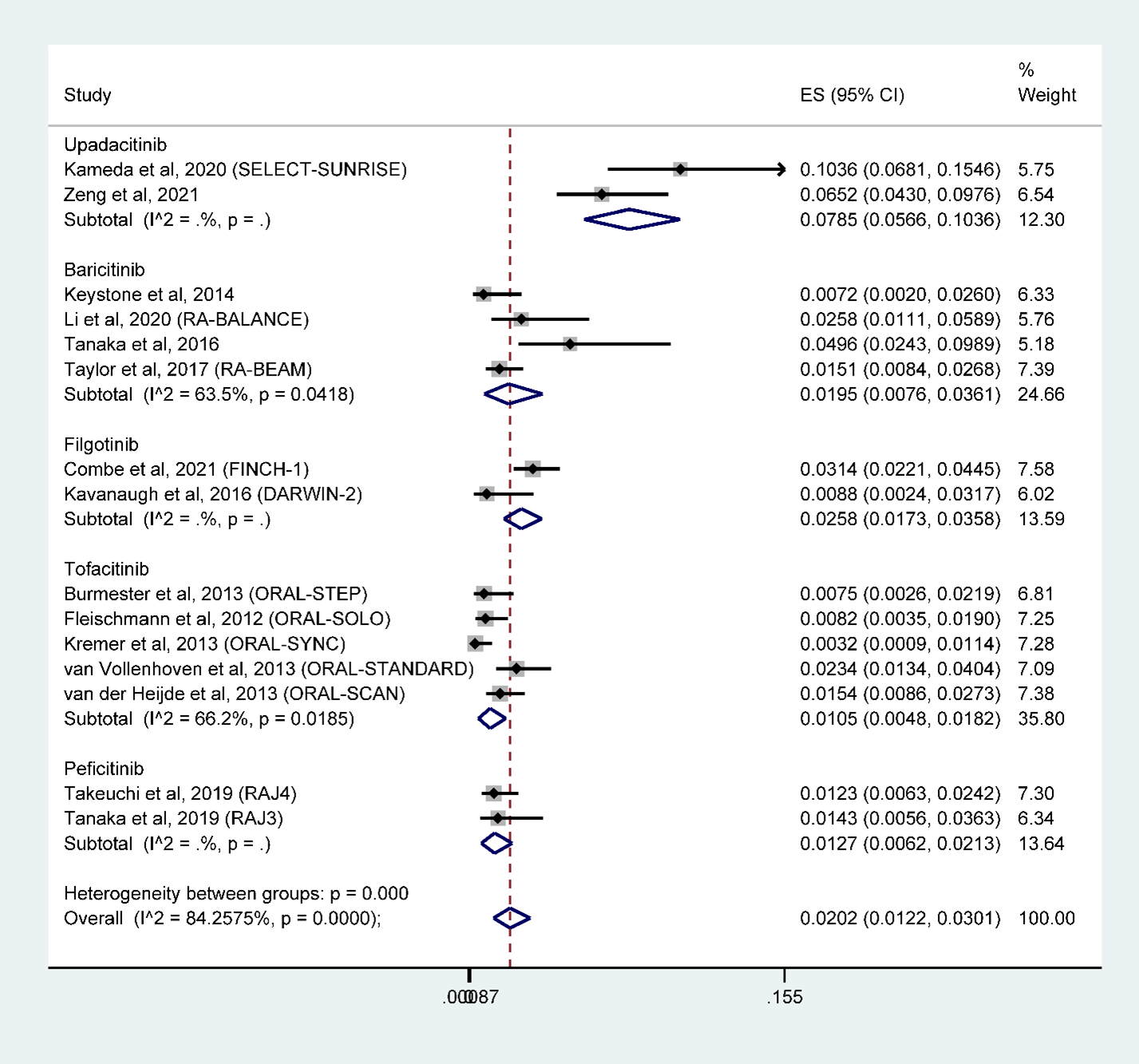

Supplement: S4 Fig — The red dotted line represents the overall pooled estimate of the cumulative incidence of severe infection in patients treated with JAKi, during follow-up extending from the time of primary study outcome assessment until the end of the study, while the weight percentages correspond to the contribution of each study to the pooled estimate. Abbreviations: CI: confidence interval; ES: effect size; JAKi: Janus-activated kinase inhibitor; RA: rheumatoid arthritis. (TIF) [file pone.0306548.s006.tif]

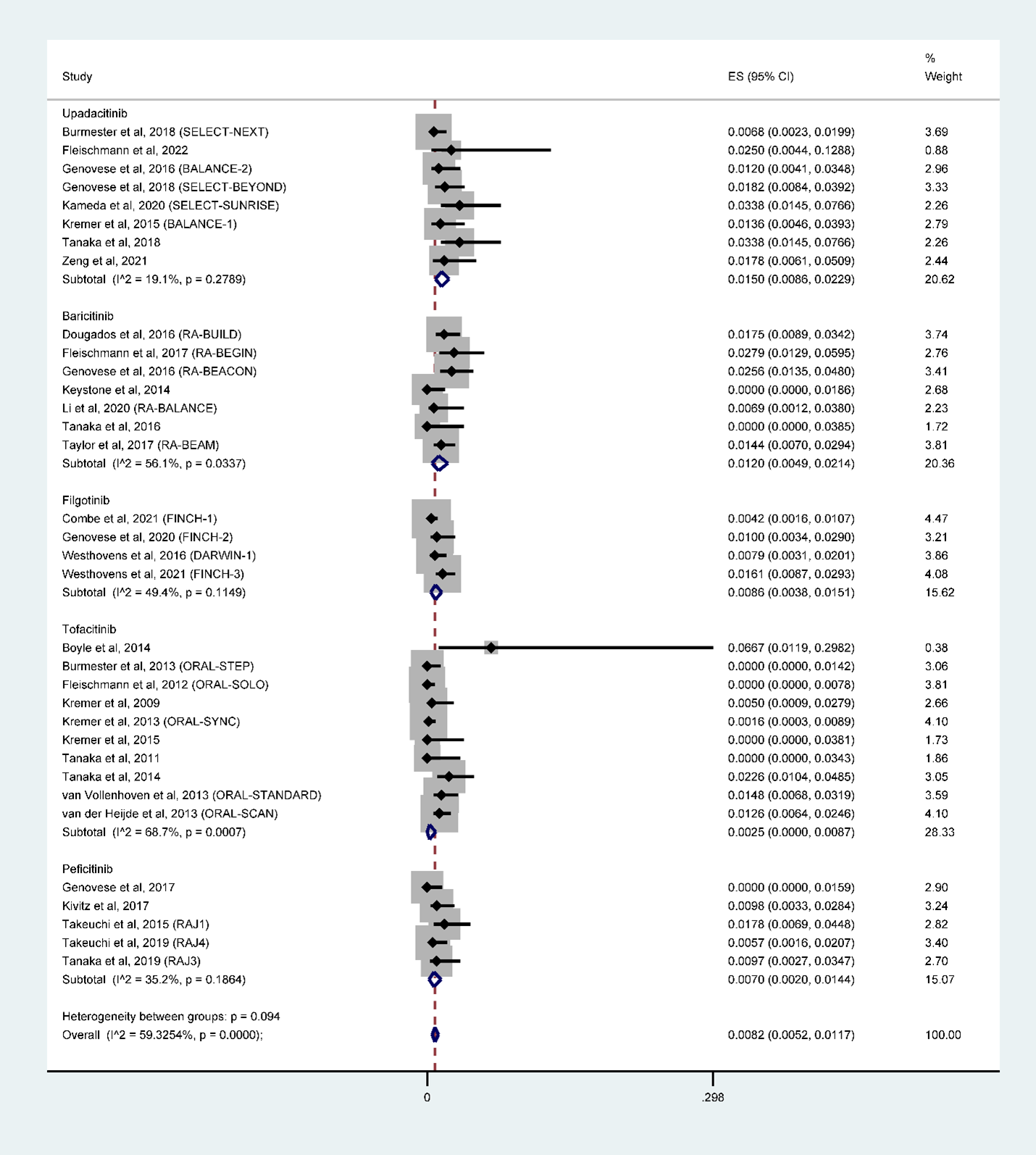

Supplement: S5 Fig — The red dotted line represents the overall pooled estimate of the cumulative incidence of opportunistic infections infection in patients treated with JAKi from study initiation until primary study outcome assessment, while the weight percentages correspond to the contribution of each study to the pooled estimate. Abbreviations: CI: confidence interval; ES: effect size; JAKi: Janus-activated kinase inhibitor; RA: rheumatoid arthritis. (TIF) [file pone.0306548.s007.tif]

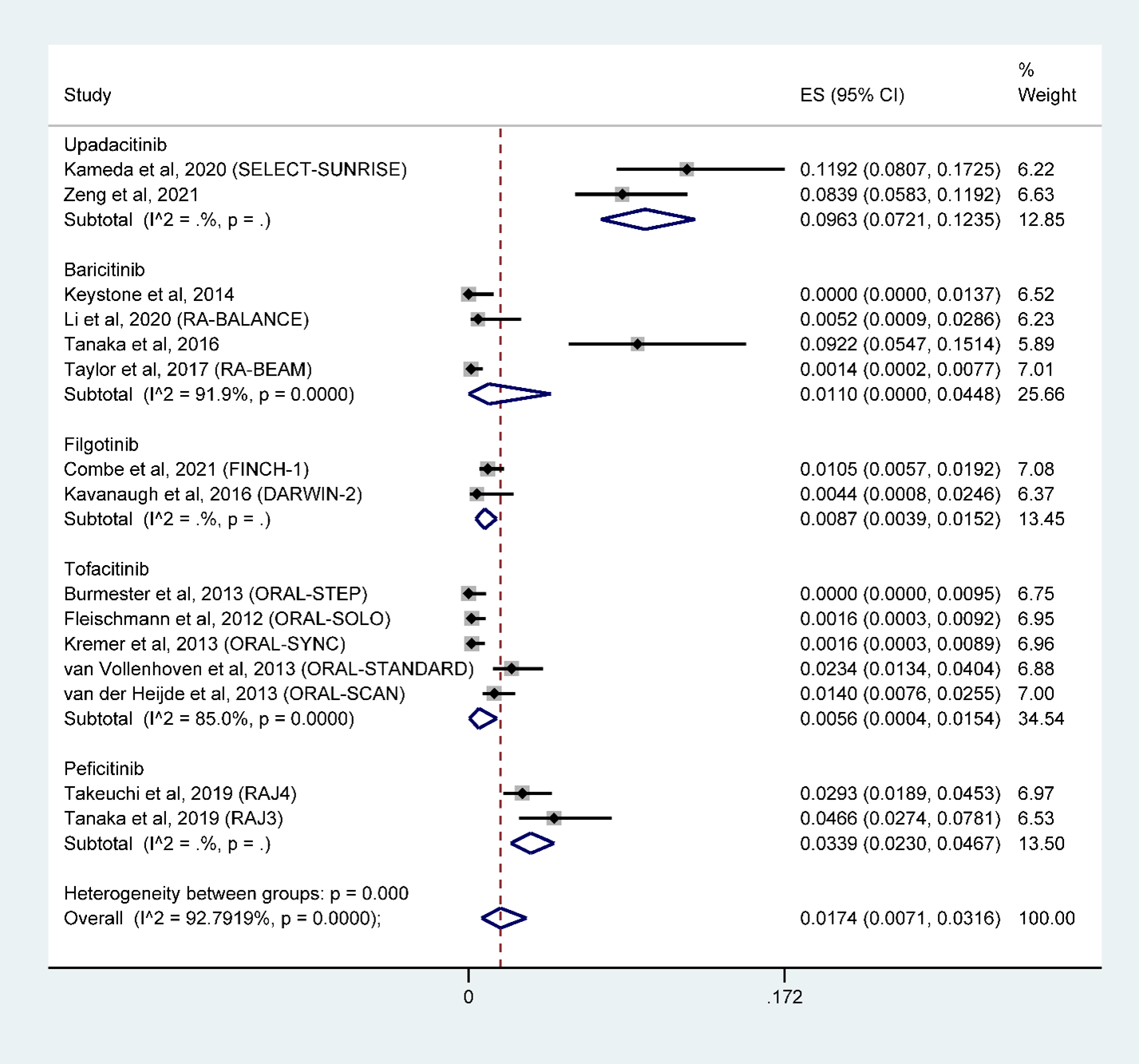

Supplement: S6 Fig — The red dotted line represents the overall pooled estimate of the cumulative incidence of opportunistic infections infection in patients treated with JAKi, during follow-up extending from the time of primary study outcome assessment until the end of the study, while the weight percentages correspond to the contribution of each study to the pooled estimate. Abbreviations: CI: confidence interval; ES: effect size; JAKi: Janus-activated kinase inhibitor; RA: rheumatoid arthritis. (TIF) [file pone.0306548.s008.tif]

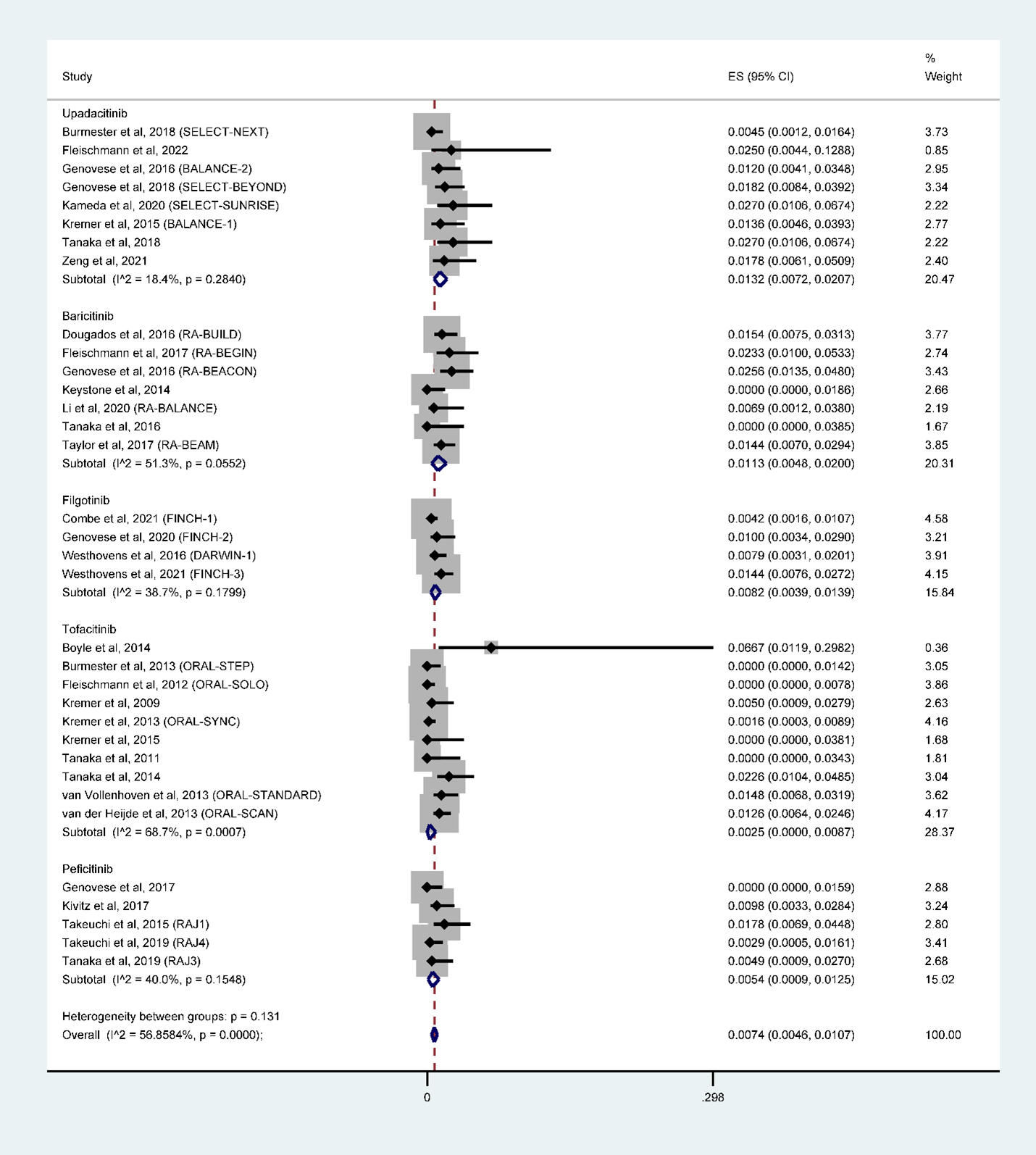

Supplement: S7 Fig — The red dotted line represents the overall pooled estimate of the cumulative incidence of herpes zoster in patients treated with JAKi from study initiation until primary study outcome assessment, while the weight percentages correspond to the contribution of each study to the pooled estimate. Abbreviations: CI: confidence interval; ES: effect size; JAKi: Janus-activated kinase inhibitor; RA: rheumatoid arthritis. (TIF) [file pone.0306548.s009.tif]

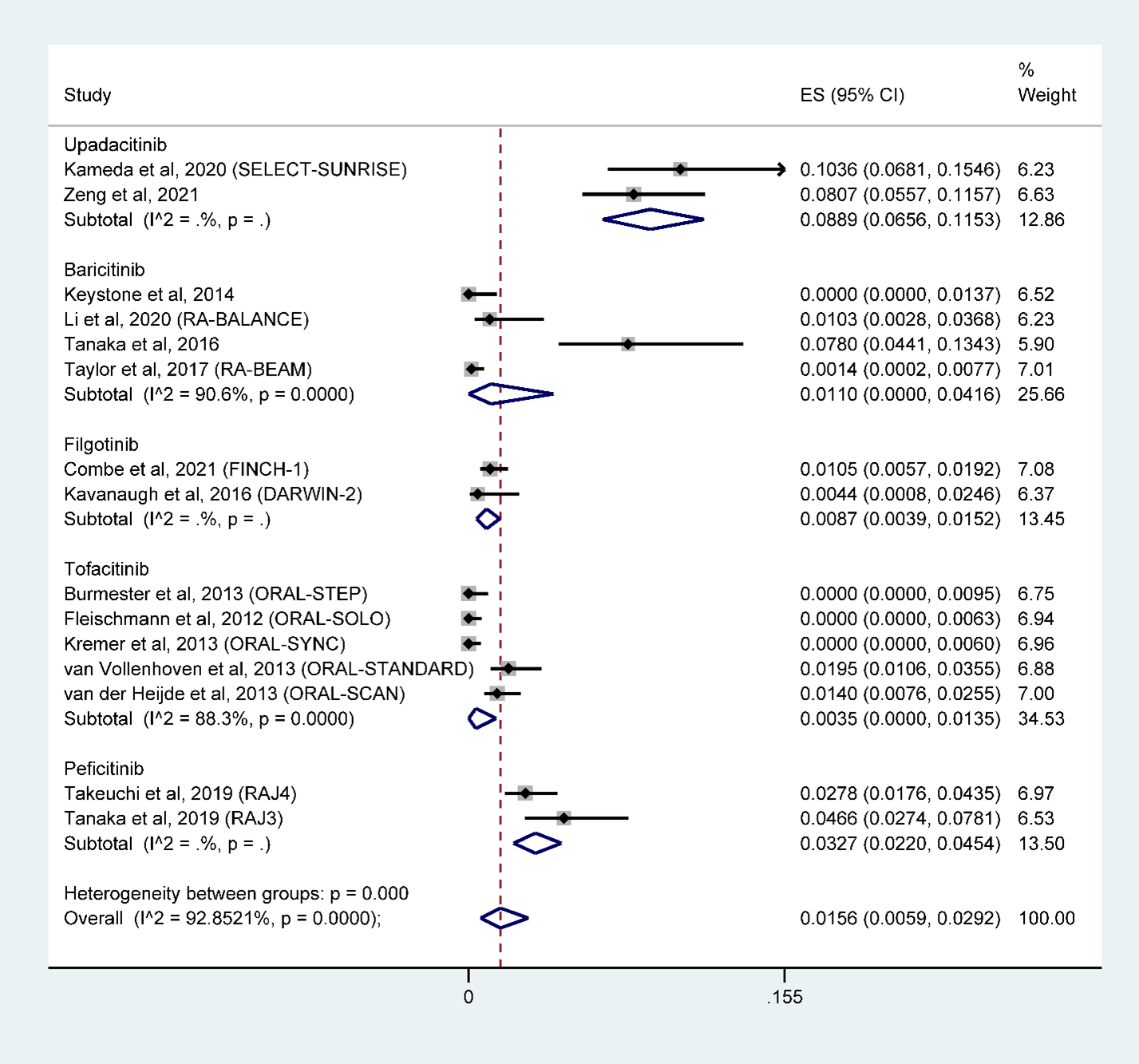

Supplement: S8 Fig — The red dotted line represents the overall pooled estimate of the cumulative incidence of herpes in patients treated with JAKi, during follow-up extending from the time of primary study outcome assessment until the end of the study, while the weight percentages correspond to the contribution of each study to the pooled estimate. Abbreviations: CI: confidence interval; ES: effect size; JAKi: Janus-activated kinase inhibitor; RA: rheumatoid arthritis. (TIF) [file pone.0306548.s010.tif]

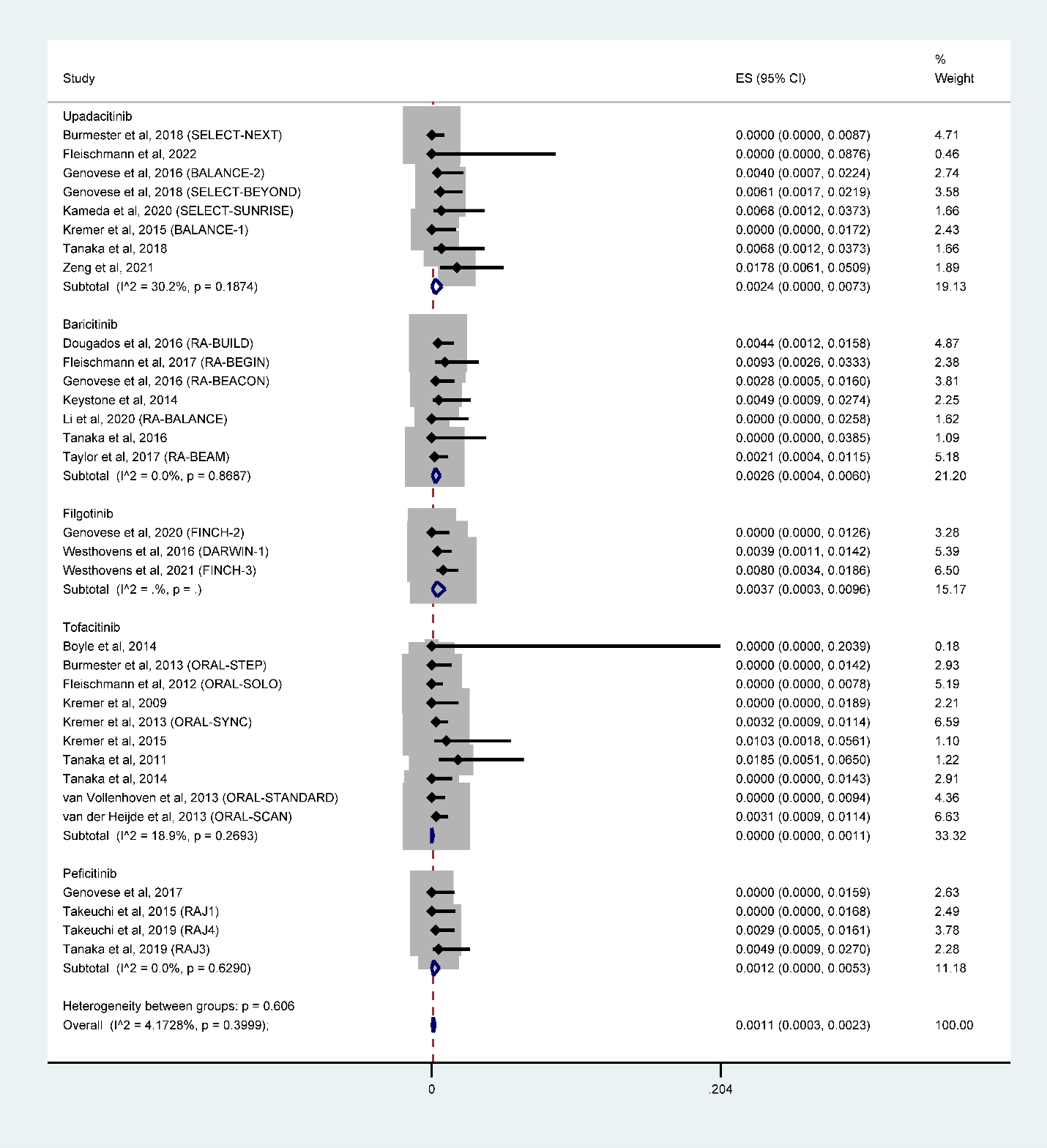

Supplement: S9 Fig — The red dotted line represents the overall pooled estimate of the cumulative incidence of pneumonia in patients treated with JAKi from study initiation until primary study outcome assessment, while the weight percentages correspond to the contribution of each study to the pooled estimate. Abbreviations: CI: confidence interval; ES: effect size; JAKi: Janus-activated kinase inhibitor; RA: rheumatoid arthritis. (TIF) [file pone.0306548.s011.tif]

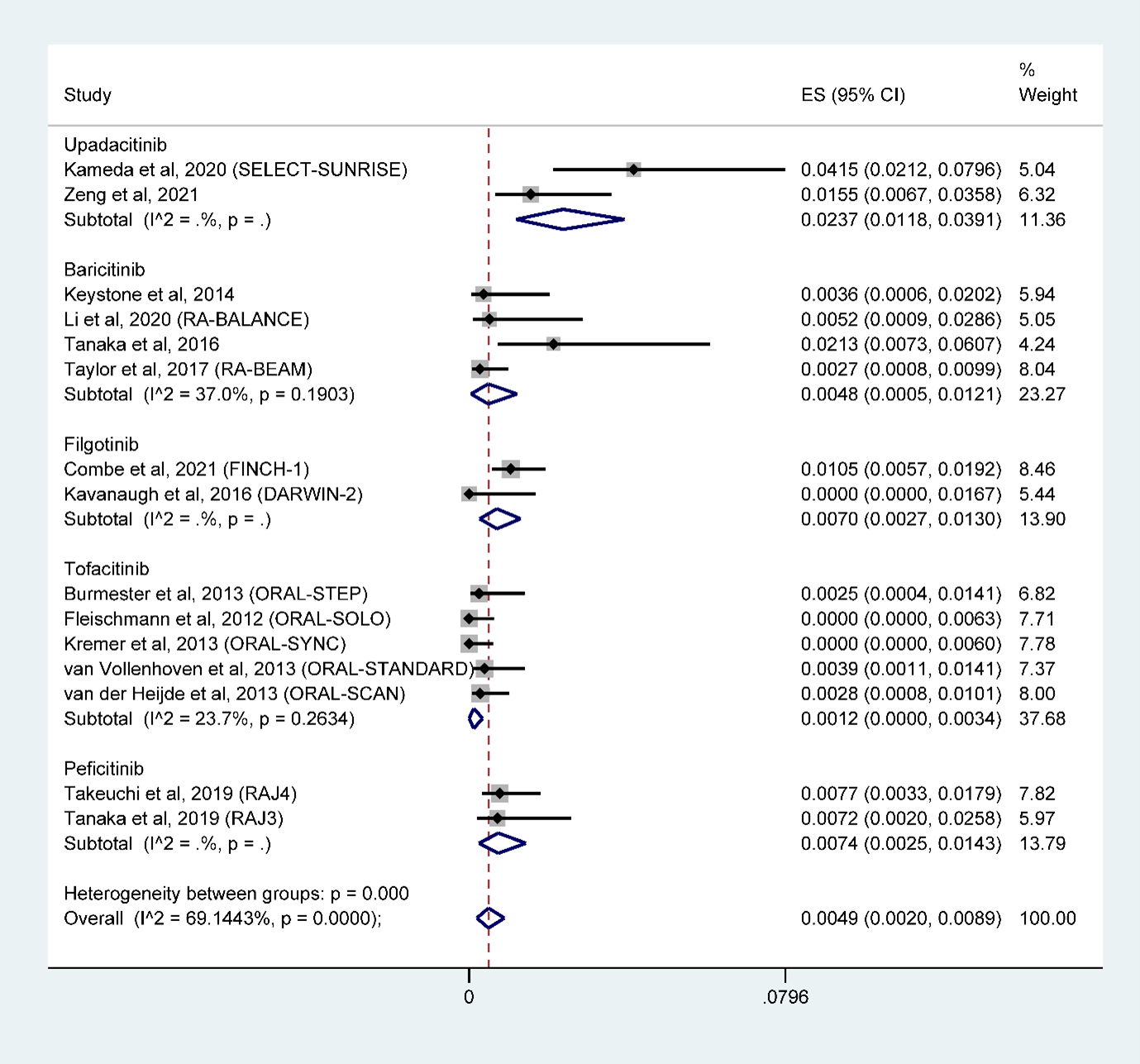

Supplement: S10 Fig — The red dotted line represents the overall pooled estimate of the cumulative incidence of pneumonia in patients treated with JAKi, during follow-up extending from the time of primary study outcome assessment until the end of the study, while the weight percentages correspond to the contribution of each study to the pooled estimate. Abbreviations: CI: confidence interval; ES: effect size; JAKi: Janus-activated kinase inhibitor; RA: rheumatoid arthritis. (TIF) [file pone.0306548.s012.tif]
