# Supplementary material for: Cumulative incidence and risk of infection in patients with rheumatoid arthritis treated with janus kinase inhibitors: A systematic review and meta-analysis
Source: PLoS One. 2024 Jul 31;19(7):e0306548. doi: 10.1371/journal.pone.0306548 (PMC11290652; doi:10.1371/journal.pone.0306548)
Supplement: S1 Table — (PDF) [file pone.0306548.s013.pdf]

S1 Table. Characteristics of the randomized controlled trials included in the analysis.

Author	Year	Study Name	NCT#	Name of drug	MOA	Duration of study*	Treatment dosing and duration	Population	Age, mean	Age, SD	Sex (female)	RA Diagnostic criteria	Concurrent MTX alone	Concurrent MTX + other csDMARD	Concurrent SLZ alone	Concurrent LFN alone	Concurrent HCQ alone	Concurrent oral glucocorticoid use	Previous use of DMARDs
Genoves et al.	2018	SELECT-BEYOND	NCT02706847	Upadacitinib	JAK1 inhibitor	12	Upadacitinib 15mg po once-daily for 12 weeks	164	56.3	11.3	137	2010 ACR and EULAR	118	19	6 (4%)	15 (9%)	7 (4%)	83	164 (100%) - bDMARDs
							Upadacitinib 30mg po once-daily for 12 weeks	165	57.3	11.6	138	2010 ACR and EULAR	124	11	9 (5%)	10 (6%)	14 (8%)	87	165 (100%) - bDMARDs
							Placebo po once-daily for 12 weeks	169	57.6	11.4	143	2010 ACR and EULAR	122	17	8 (5%)	13 (8%)	11 (7%)	74	169 (100%) - bDMARDs
Fleischmann et al.	2017	RA-BEGIN	NCT01711359	Baricitinib	JAK1/JAK2 inhibitor	52	Baricitinib 4mg once-daily po + MTX up to 20mg/week for 52 weeks	215	49	14	156	2010 ACR and EULAR	215	0	0	0	0	83	0
							MTX up to 20mg/week for 52 weeks	210	51	13	148	2010 ACR and EULAR	210	0	0	0	0	76	0
							Baricitinib 4mg once-daily po for 52 weeks	159	51	13	121	2010 ACR and EULAR	159	0	0	0	0	47	0
Westhovens et al.	2021	FINCH 3	NCT02886728	Filgotinib	JAK1 inhibitor	52	Filgotinib 200mg po once-daily + MTX up to 15mg/week for 52 weeks	416	53	13.8	325	2010 ACR and EULAR	416	0	0	0	35 (8%)	143	97 (23.3%)
							Filgotinib 100mg po once-daily + MTX up to 15mg/week for 52 weeks	207	54	12.6	158	2010 ACR and EULAR	207	0	0	0	24 (12%)	88	48 (23.2%)

							Filgotinib 200mg po once-daily for 52 weeks	210	52	13.9	166	2010 ACR and EULAR	0	0	0	0	17 (8%)	89	46 (21.9%)
							MTX up to 15mg/week for 52 weeks	416	53	13.7	312	2010 ACR and EULAR	416	0	0	0	42 (10%)	174	94 (22.6%)
Kremer et al.	2013	ORAL-SYNC	NCT00856544	Tofacitinib	JAK inhibitor	12	Tofacitinib 5mg twice daily until week 12	315	52.7	11.7	264	1987 ACR Revised Criteria	250	NA	NA	NA	NA	195	232 (73.7%) (DMARDs other than MTX), 273 (86.7%) (MTX)
							Tofacitinib 10 mg twice daily until week 12	318	51.9	11.8	258	1987 ACR Revised Criteria	251	NA	NA	NA	NA	182	242 (76.1%)(DMARDs other than MTX), 263 (82.7%) (MTX)
							Placebo twice daily until week 12	159	52.1	11	123	1987 ACR Revised Criteria	125	NA	NA	NA	NA	94	117 (73.6%) (DMARDs other than MTX), 132 (83%)
Kremer et al.	2015		NCT01484561	Tofacitinib	JAK inhibitor	21.5	Tofacitinib 10mg twice daily for up to 50 days, followed by placebo twice daily for up to 36 days	97	51.86	12.68	75	1987 ACR Revised Criteria	NA	NA	NA	NA	NA	NA	94 (96.9%) (DMARD, unspecified), 87 (89.7%) (MTX)
							Placebo for up to 86 days	51	47.25	11.84	36	1987 ACR Revised Criteria	NA	NA	NA	NA	NA	NA	47 (92.2%) (DMARD, unspecified), 47 (92.2%) (MTX)
Zeng et al.	2021		NCT02955212	Upadacitinib	JAK1 inhibitor	12	Upadacitinib 15mg once-daily for 12 weeks	169	51.7	11.4	139	2010 ACR and EULAR	71	41	NA	NA	NA	112	3 (1.8%) (bDMARD)
							Placebo once-daily for 12 weeks	169	51.7	10.6	135	2010 ACR and EULAR	79	32	NA	NA	NA	108	5 (3%) (bDMARD)
Taylor et al.	2017	RA-BEAM	NCT01710358	Baricitinib	JAK1/JAK2 inhibitor	24	Baricitinib 4mg po once daily for 24 weeks	487	54	2	375	≥6 tender joints of 68 examined, ≥6 swollen joints of 66 examined, and a high-sensitivity serum C-reactive protein level of ≥6 mg per liter	413	74	NA	NA	NA	275	One csDMARD (243) (50%), two csDMARDs (138) (28%), ≥3 csDMARDs (106) (22%)
							Placebo po once daily for 24 weeks	488	53	2	382	≥6 tender joints of 68 examined, ≥6 swollen joints of 66 examined, and a high-sensitivity serum C-reactive protein level of ≥6 mg per liter	398	89	NA	NA	NA	290	One csDMARD (204) (42%), two csDMARDs (169) (35%), ≥3 csDMARDs (114) (23%)

Combe et al.	2021	FINCH 1	NCT02 889796	Filgotinib	JAK1 inhibitor	24	Filgotinib 100mg po once-daily for 24 weeks	475	52	12.8	379	2010 ACR and EULAR	475	NA	NA	NA	64 (13.5%)	229	bDMARD (17) (3.6%)
							Filgotinib 200mg po once-daily for 24 weeks	480	53	12.6	399	2010 ACR and EULAR	480	NA	NA	NA	59 (12.3%)	229	bDMARD (16) (3.3%)
							Placebo for 24 weeks	475	53	12.8	391	2010 ACR and EULAR	475	NA	NA	NA	63 (13.3%)	217	bDMARD (6) (1.3%)
Takeuchi et al.	2019	RAJ4	NCT02 305849	Peficitinib	JAK inhibitor	12	Peficitinib 150mg once-daily for 12 weeks	174	56.2	11.6	125	2010 ACR and EULAR	174	NA	NA	NA	NA	NA	bDMARD (27) (15.5%), non-bDMARDs, except MTX (95) (54.6%)
							Peficitinib 100mg once-daily for 12 weeks	174	58.5	10.8	118	2010 ACR and EULAR	174	NA	NA	NA	NA	NA	bDMARD (33) (19.9%), non-bDMARDs, except MTX (105) (60.3%)
							Placebo once-daily for 12 weeks	170	55.3	12.1	121	2010 ACR and EULAR	170	NA	NA	NA	NA	NA	bDMARD (38) (22.4%), non-bDMARDs, except MTX (97) (57.1%)
Genovese et al.	2016	RA-BEACON	NCT01 721044	Baricitinib	JAK1/2 inhibitor	24	Baricitinib 4mg once-daily for 24 weeks	177	56	11	149	≥6 tender joints of 68 examined, ≥6 swollen joints of 66 examined, and a serum C-reactive protein level of ≥3 mg per liter	150	NA	NA	NA	NA	96	one bDMARD (71) (40%), two bDMARDs (58) (33%), ≥3 bDMARDs (45) (25%)
							Baricitinib 2mg once-daily for 24 weeks	174	55	11	137	≥6 tender joints of 68 examined, ≥6 swollen joints of 66 examined, and a serum C-reactive protein level of ≥3 mg per liter	141	NA	NA	NA	NA	92	one bDMARD (69) (40%), two bDMARDs (55) (32%), ≥3 bDMARDs (50) (29%)
							Placebo once-daily for 24 weeks	176	56	11	145	≥6 tender joints of 68 examined, ≥6 swollen joints of 66 examined, and a serum C-reactive protein level of ≥3 mg per liter	143	NA	NA	NA	NA	116	one bDMARD (81) (46%), two bDMARDs (47) (27%), ≥3 bDMARDs (47) (27%)
Dougados et al.	2016	RA-BUILD	NCT01 721057	Baricitinib	JAK1/2 inhibitor	24	Baricitinib 4mg once-daily for 24 weeks	227	52	12	187	≥6/68 tender and ≥6/66 swollen joints; serum high sensitivity C-reactive protein (CRP) ≥3.6mg/L	173	51	22 (10%)	29 (13%)	54 (24%)	115	one bDMARD (98) (43%), two bDMARDs (68) (30%), ≥3 bDMARDs (60) (26%)
							Baricitinib 2mg once-daily for 24 weeks	229	52	12	184	≥6/68 tender and ≥6/66 swollen joints; serum high sensitivity C-reactive protein (CRP) ≥3.6mg/L	168	51	28 (12%)	21 (9%)	63 (28%)	117	one bDMARD (104) (45%), two bDMARDs (61) (27%), ≥3

																			bDMARDs (61) (27%)
							Placebo once-daily for 24 weeks	228	51	13	189	≥6/68 tender and ≥6/66 swollen joints; serum high sensitivity C-reactive protein (CRP) ≥3.6mg/L	109	52	23 (10%)	28 (12%)	54 (24%)	114	one bDMARD (96) (42%), two bDMARDs (81) (36%), ≥3 bDMARDs (50) (22%)
Burmester et al.	2018	SELECT-NEXT	NCT02675426	Upadacitinib	JAK1 inhibitor	12	Upadacitinib 30mg once-daily for 12 weeks	219	55.8	11.3	172	2010 ACR and EULAR	136	39	NA	NA	NA	103	bDMARD (28) (13%)
							Upadacitinib 15mg once-daily for 12 weeks	221	55.3	11.5	182	2010 ACR and EULAR	122	47	NA	NA	NA	96	bDMARD (27) (12%)
							Placebo once-daily for 12 weeks	221	56	12.2	166	2010 ACR and EULAR	141	49	NA	NA	NA	106	bDMARD (29) (13%)
Tanaka et al.	2019	RAJ3	NCT02308163	Peficitinib	JAK inhibitor	12	Peficitinib 150mg once-daily for 12 weeks	102	55	12.8	78	1987 ACR Revised Criteria and 2010 ACR and EULAR	62	NA	NA	NA	NA	NA	bDMARD (0), non-bDMARD (89) (87.3%)
							Peficitinib 100mg once-daily for 12 weeks	104	54.1	12.2	77	1987 ACR Revised Criteria and 2010 ACR and EULAR	63	NA	NA	NA	NA	NA	bDMARD (0), non-bDMARD (90) (86.5%)
							Placebo once-daily for 12 weeks	101	56.3	11.7	73	1987 ACR Revised Criteria and 2010 ACR and EULAR	57	NA	NA	NA	NA	NA	bDMARD (0), non-bDMARD (90) (89.1%)
Genovese et al.	2020	FINCH-2	NCT02873936	Filgotinib	JAK inhibitor	24	Filgotinib 200mg once-daily for 24 weeks	147	56	12.5	120	6 or more swollen joints (swollen joint count [SJC] of 66 joints [SJC66]), and 6 or more tender joints (tender joint count [TJC] of 68 joints [TJC68]) at both	124	NA	NA	NA	NA	68	<3 bDMARDs (110) (74.8%), ≥3 bDMARDs (37) (25.2%)

												screening and baseline, and a serum C-reactive protein (CRP) level of 4 mg/L or greater							
							Filgotinib 100mg once-daily for 24 weeks	153	55	12	119	6 or more swollen joints (swollen joint count [SJC] of 66 joints [SJC66]), and 6 or more tender joints (tender joint count [TJC] of 68 joints [TJC68]) at both screening and baseline, and a serum C-reactive protein (CRP) level of 4 mg/L or greater	127	NA	NA	NA	NA	68	<3 bDMARDs (119) (77.8%), ≥3 bDMARDs (34) (22.2%)
							Placebo once-daily for 24 weeks	148	56	12.1	121	6 or more swollen joints (swollen joint count [SJC] of 66 joints [SJC66]), and 6 or more tender joints (tender joint count [TJC] of 68 joints [TJC68]) at both screening and baseline, and a serum C-reactive protein (CRP) level of 4 mg/L or greater	116	NA	NA	NA	NA	71	<3 bDMARDs (114) (77%), ≥3 bDMARDs (34) (23%)
Westhovens et al.	2016	DARWIN-1 (only for incidence assessment- after week 12, crossover between placebo and filgotinib groups occurred, and safety events were reported for weeks 0-24, making inferences about the risk of infection unreliable)	NCT01888874	Filgotinib	JAK1 inhibitor	24	Filgotinib 200mg daily for 12 weeks	170	54.5	1.3	144	2010 ACR and EULAR	170	0	0	0	0	99	bDMARD (18) (10.6%)
							Filgotinib 100mg daily for 12 weeks	170	53.5	1.35	130	2010 ACR and EULAR	170	0	0	0	0	104	bDMARD (12) (7.1%)
							Filgotinib 50mg daily for 12 weeks	168	52.5	1.45	137	2010 ACR and EULAR	168	0	0	0	0	99	bDMARD (12) (7.1%)
							Placebo daily for 12 weeks	86	52	1.4	70	2010 ACR and EULAR	86	0	0	0	0	50	bDMARD (8) (9.3%)
Kavanaugh et al.	2016	DARWIN-2	NCT01894516	Filgotinib	JAK1 inhibitor	12	Filgotinib 200mg once-daily for 12 weeks	69	52	1.4	60	2010 ACR and EULAR	0	0	0	0	NA	47	MTX (58) (84.1%),

																		bDMARD (5) (7.2%)
						Filgotinib 100mg once-daily for 12 weeks	70	53	1.4	53	2010 ACR and EULAR	0	0	0	0	NA	50	MTX (59) (84.3%), bDMARD (4) (5.7%)
						Filgotinib 50mg once-daily for 12 weeks	72	52	1.6	62	2010 ACR and EULAR	0	0	0	0	NA	47	MTX (61) (84.7%), bDMARD (7) (9.7%)
						Placebo once-daily for 12 weeks	72	52	1.4	56	2010 ACR and EULAR	0	0	0	0	NA	45	MTX (60) (83.3%), bDMARD (3) (4.2%)
Keystone et al.	2014	NCT01 185353	Baricitinib	JAK1/2 inhibitor	12	Baricitinib 8mg once-daily for 12 weeks	50	53	11	41	Presence of eight or more tender and eight or more swollen joints (from a 68/66-joint count) and either a high sensitivity C-reactive protein (CRP) level >1.2× the upper limit of normal (ULN;>3.6mg/L) or an erythrocyte sedimentation rate (ESR)>28mm/h.	50	NA	9 (17%)	NA	7 (13%)	20	NA
						Baricitinib 4mg once-daily for 12 weeks	52	53	10	37	Presence of eight or more tender and eight or more swollen joints (from a 68/66-joint count) and either a high sensitivity C-reactive protein (CRP) level >1.2× the upper limit of normal (ULN;>3.6mg/L) or an erythrocyte sedimentation rate (ESR)>28mm/h.	51	NA	9 (17%)	NA	7 (13%)	20	NA
						Baricitinib 2mg once-daily for 12 weeks	52	51	13	44	Presence of eight or more tender and eight or more swollen joints (from a 68/66-joint count) and either a high sensitivity C-reactive protein (CRP) level >1.2× the upper limit of normal (ULN;>3.6mg/L) or an erythrocyte sedimentation rate (ESR)>28mm/h.	52	NA	7 (13%)	NA	11 (21%)	27	NA
						Baricitinib 1mg once-daily for 12 weeks	49	53	11	42	Presence of eight or more tender and eight or more swollen joints (from a 68/66-joint count) and either a high sensitivity C-reactive protein (CRP) level >1.2× the upper limit of normal	49	NA	4 (8%)	NA	11 (22%)	21	NA

												(ULN;>3.6mg/L) or an erythrocyte sedimentation rate (ESR)>28mm/h.							
							Placebo once-daily for 12 weeks	98	49	12	43	Presence of eight or more tender and eight or more swollen joints (from a 68/66-joint count) and either a high sensitivity C-reactive protein (CRP) level >1.2× the upper limit of normal (ULN;>3.6mg/L) or an erythrocyte sedimentation rate (ESR)>28mm/h.	98	NA	14 (14%)	NA	16 (16%)	51	NA
van Vollenhoven et al.	2013	ORAL-STANDARD	NCT00853385	Tofacitinib	JAK inhibitor	12	Tofacitinib 10mg twice daily for 12 weeks	201	52.9	11.8	168	1987 ACR Revised Criteria	201	NA	NA	NA	NA	129	TNF-alpha inhibitor (14) (7%), non-TNF-alpha bDMARD (4) (2%), DMARD other than MTX (115) (57.2%)
							Tofacitinib 5mg twice daily for 12 weeks	204	53	11.9	174	1987 ACR Revised Criteria	204	NA	NA	NA	NA	126	TNF-alpha inhibitor (12) (5.9%), non-TNF-alpha bDMARD (2) (1%), DMARD other than MTX (109) (53.4%)
							Placebo twice daily for 12 weeks	108	53.5	13.7	82	1987 ACR Revised Criteria	108	NA	NA	NA	NA	72	TNF-alpha inhibitor (9) (8.3%), non-TNF-alpha bDMARD (6) (5.6%), DMARD other than MTX (59) (54.6%)
Strand et al.	2012	ORAL-SOLO	NCT00814307	Tofacitinib	JAK inhibitor	12	Tofacitinib 10mg twice-daily for 12 weeks	245	52.4	11.7	216	1987 ACR Revised Criteria	NA	NA	NA	NA	41 (16.7%)	148	TNF-alpha inhibitor (41) (16.7%), non-TNF-alpha bDMARD (19) (7.8%), MTX (207) (84.5%), csDMARD other than MTX (141) (57.6%)
							Tofacitinib 5mg twice-daily for 12 weeks	243	52.2	11.5	207	1987 ACR Revised Criteria	NA	NA	NA	NA	45 (18.5%)	139	TNF-alpha inhibitor (34) (14%), non-TNF-alpha bDMARD (12) (4.9%),

																			MTX (209) (86%), csDMARD other than MTX (132) (54.3%)
							Placebo twice-daily for 12 weeks	122	49.7	12.4	105	1987 ACR Revised Criteria	NA	NA	NA	NA	15 (12.3%)	77	TNF-alpha inhibitor (24) (19.7%), non-TNF-alpha bDMARD (10) (8.2%), MTX (102) (83.6%), csDMARD other than MTX (74) (60.7%)
Burmester et al.	2013	ORAL-STEP	NCT00960440	Tofacitinib	JAK inhibitor	12	Tofacitinib 10mg twice-daily for 12 weeks	134	55.1	11.3	116	1987 ACR Revised Criteria	134	NA	NA	NA	7 (5.2%)	81	TNF-alpha inhibitors (132) (98.5%), non-TNF-alpha bDMARDs (11) (8.2%), csDMARDs other than MTX (37) (27.6%)
							Tofacitinib 5mg twice-daily for 12 weeks	133	55.4	11.5	113	1987 ACR Revised Criteria	131	NA	NA	NA	12 (9%)	85	TNF-alpha inhibitors (132) (99.2%), non-TNF-alpha bDMARDs (21) (15.8%), csDMARDs other than MTX (53) (39.8%)
							Placebo twice-daily for 12 weeks	132	54.4	11.3	106	1987 ACR Revised Criteria	132	NA	NA	NA	5 (3.8%)	83	TNF-alpha inhibitors (132) (100%), non-TNF-alpha bDMARDs (14) (10.6%), csDMARDs other than MTX (33) (25%)
van der Heijde	2013	ORAL-SCAN	NCT00847613	Tofacitinib	JAK inhibitor	12	Tofacitinib 10mg twice-daily for 12 weeks	316	52	11.4	273	1987 ACR Revised Criteria	315	NA	NA	NA	NA	NA	TNF-alpha inhibitors (50) (15.8%), non-TNF-alpha bDMARDs (15) (4.7%), csDMARDs other than MTX (192) (60.8%)
							Tofacitinib 5mg twice-daily for 12 weeks	321	53.7	11.6	269	1987 ACR Revised Criteria	321	NA	NA	NA	NA	NA	TNF-alpha inhibitors (62) (19.3%), non-TNF-alpha bDMARDs (17)

																			(5.3%), csDMARDs other than MTX (193) (60.1%)
							Placebo twice-daily for 12 weeks	160	52.7	11.7	137	1987 ACR Revised Criteria	160	NA	NA	NA	NA	NA	TNF-alpha inhibitors (14) (8.8%), non-TNF-alpha bDMARDs (4) (2.5%), csDMARDs other than MTX (108) (67.5%)
Kremer et al.	2009		NCT00147498	Tofacitinib	JAK inhibitor	6	Tofacitinib 30mg twice daily for 6 weeks	69	51.1	10.6	60	1987 ACR Revised Criteria	0	0	0	0	0	44	MTX (54) (78.3%), TNF-alpha inhibitors (1) (1.4%)
							Tofacitinib 15mg twice daily for 6 weeks	69	51.8	13	58	1987 ACR Revised Criteria	0	0	0	0	0	43	MTX (48) (69.6%), TNF-alpha inhibitors (2) (2.9%)
							Tofacitinib 5mg twice daily for 6 weeks	61	47.9	10.8	53	1987 ACR Revised Criteria	0	0	0	0	0	39	MTX (47) (77%), TNF-alpha inhibitors (3) (4.9%)
							Placebo twice daily for 6 weeks	65	51.3	12.1	55	1987 ACR Revised Criteria	0	0	0	0	0	40	MTX (52) (80%), TNF-alpha inhibitors (0)
Takeuchi et al.	2015	RAJ1	NCT01649999	Peficitinib	JAK inhibitor	12	Peficitinib 150mg once-daily for 12 weeks	58	51.6	12.1	51	1987 ACR Revised Criteria	0	0	0	0	0	NA	TNF-alpha inhibitors (16) (27.6%), MTX (54) (93.1%)
							Peficitinib 100mg once-daily for 12 weeks	55	52.1	12.1	42	1987 ACR Revised Criteria	0	0	0	0	0	NA	TNF-alpha inhibitors (14) (25.5%), MTX (47) (85.5%)
							Peficitinib 50mg once-daily for 12 weeks	57	54.2	11.6	46	1987 ACR Revised Criteria	0	0	0	0	0	NA	TNF-alpha inhibitors (14) (24.6%), MTX (51) (89.5%)
							Peficitinib 25mg once-daily for 12 weeks	55	52.9	9.5	46	1987 ACR Revised Criteria	0	0	0	0	0	NA	TNF-alpha inhibitors (11) (20%), MTX (49) (89.1%)
							Placebo once-daily for 12 weeks	56	54.2	12.1	43	1987 ACR Revised Criteria	0	0	0	0	0	NA	TNF-alpha inhibitors (16) (28.6%), MTX (50) (89.3%)
Kameda et al.	2020	SELECT-SUNRISE	NCT02720523	Upadacitinib	JAK1 inhibitor	12	Upadacitinib 30mg once-daily for 12 weeks	50	54.7	12.2	43	2010 ACR and EULAR	37	7	NA	NA	NA	24	bDMARD (3) (6%)
							Upadacitinib 15mg once-daily for 12 weeks	49	56	12.5	36	2010 ACR and EULAR	28	12	NA	NA	NA	28	bDMARD (6) (12.2%)

						Upadacitinib 7.5mg once-daily for 12 weeks	49	55.8	11	34	2010 ACR and EULAR	25	13	NA	NA	NA	26	bDMARD (5) (10.2%)
						Placebo once-daily for 12 weeks	49	54.3	13	42	2010 ACR and EULAR	29	14	NA	NA	NA	24	bDMARD (3) (6.1%)
Tanaka et al.	2016	NCT01 469013	Baricitinib	JAK1/2 inhibitor	12	Baricitinib 8mg once-daily for 12 weeks	24	53.6	11.3	17	2010 ACR and EULAR	24	NA	NA	NA	NA	13	NA
						Baricitinib 4mg once-daily for 12 weeks	24	57.5	10.4	19	2010 ACR and EULAR	24	NA	NA	NA	NA	18	NA
						Baricitinib 2mg once-daily for 12 weeks	24	56.1	11.5	21	2010 ACR and EULAR	24	NA	NA	NA	NA	13	NA
						Baricitinib 1mg once-daily for 12 weeks	24	52.7	12.8	22	2010 ACR and EULAR	24	NA	NA	NA	NA	12	NA
						Placebo once-daily for 12 weeks	49	51.1	12	39	2010 ACR and EULAR	49	NA	NA	NA	NA	29	NA
Kivitz et al.	2017	NCT01 554696	Peficitinib	JAK1/2 inhibitor	12	Peficitinib 150mg once-daily + MTX for 12 weeks	78	54.2	12.5	64	1987 ACR Revised Criteria	78	NA	4 (5.1%)	NA	7 (9%)	NA	LFN (3) (3.8%), TNF-alpha inhibitor (19) (24.4%)
						Peficitinib 100mg once-daily + MTX for 12 weeks	84	54.5	12.8	68	1987 ACR Revised Criteria	84	NA	3 (3.6%)	NA	8 (9.5%)	NA	LFN (5) (6%), TNF-alpha inhibitor (22) (26.2%)
						Peficitinib 50mg once-daily + MTX for 12 weeks	78	52.3	12.6	65	1987 ACR Revised Criteria	78	NA	3 (3.8%)	NA	6 (7.7%)	NA	LFN (2) (2.6%), TNF-alpha inhibitor (23) (29.5%)
						Peficitinib 25mg once-daily + MTX for 12 weeks	66	52.8	11.9	55	1987 ACR Revised Criteria	66	NA	3 (4.5%)	NA	10 (15.2%)	NA	LFN (2) (3%), TNF-alpha inhibitor (17) (25.8%)

							MTX once-daily for 12 weeks	72	52.6	12.2	63	1987 ACR Revised Criteria	72	NA	7 (9.7%)	NA	4 (5.6%)	NA	LFN (2) (2.8%), TNF-alpha inhibitor (19) (26.4%)
Genovese et al.	2016	BALANCE-2	NCT02066389	Upadacitinib	JAK1 inhibitor	12	Upadacitinib 24mg once-daily for 12 weeks	49	56	12	42	1987 ACR Revised Criteria	49	NA	NA	NA	NA	5	non-MTX DMARDs (12) (24%)
							Upadacitinib 18mg twice-daily for 12 weeks	50	55	14	42	1987 ACR Revised Criteria	50	NA	NA	NA	NA	6	non-MTX DMARDs (5) (10%)
							Upadacitinib 12mg twice-daily for 12 weeks	50	56	12	41	1987 ACR Revised Criteria	50	NA	NA	NA	NA	16	non-MTX DMARDs (11) (22%)
							Upadacitinib 6mg twice-daily for 12 weeks	50	55	12	34	1987 ACR Revised Criteria	50	NA	NA	NA	NA	16	non-MTX DMARDs (12) (24%)
							Upadacitinib 3mg twice-daily for 12 weeks	50	53	12	40	1987 ACR Revised Criteria	50	NA	NA	NA	NA	10	non-MTX DMARDs (6) (12%)
							Placebo twice-daily for 12 weeks	50	55	12	38	1987 ACR Revised Criteria	50	NA	NA	NA	NA	8	non-MTX DMARDs (7) (14%)
Tanaka et al.	2014		NCT00687193	Tofacitinib	JAK inhibitor	12	Tofacitinib 15mg twice-daily for 12 weeks	54	53.6	12.5	44	1987 ACR Revised Criteria	0	0	0	0	0	0	NA
							Tofacitinib 10mg twice-daily for 12 weeks	53	54.7	10.8	44	1987 ACR Revised Criteria	0	0	0	0	0	0	NA
							Tofacitinib 5mg twice-daily for 12 weeks	52	52.6	10.9	44	1987 ACR Revised Criteria	0	0	0	0	0	0	NA
							Tofacitinib 3mg twice-daily for 12 weeks	53	52.8	11.6	47	1987 ACR Revised Criteria	0	0	0	0	0	0	NA
							Tofacitinib 1mg twice-daily for 12 weeks	53	53.3	9.9	42	1987 ACR Revised Criteria	0	0	0	0	0	0	NA
							Placebo twice-daily for 12 weeks	52	53.3	11.4	43	1987 ACR Revised Criteria	0	0	0	0	0	0	NA
Boyle et al.	2014		NCT00976599	Tofacitinib	JAK inhibitor	4	Tofacitinib 10mg twice-daily for 4 weeks	15	53.5	9.2	14	1987 ACR Revised Criteria	15	NA	NA	NA	NA	NA	NA
							Placebo twice-daily for 4 weeks	14	53.1	14.3	12	1987 ACR Revised Criteria	14	NA	NA	NA	NA	NA	NA
Li et al.	2020	RA-BALANCE** (only for incidence because rescue therapy limits comparisons between treatment and control groups)	NCT02265705	Baricitinib	JAK1/2 inhibitor	24	Baricitinib 2mg or 4mg once-daily for 52 weeks	145	49.5	10.6	233	2010 ACR and EULAR	145	NA	NA	NA	NA	NA	NA
Kremer et al.	2015	BALANCE-1	NCT01960855	Upadacitinib	JAK1 inhibitor	12	Upadacitinib 18mg twice-daily for 12 weeks	55	57	12	42	1987 ACR Revised Criteria	55	NA	NA	NA	NA	NA	TNF-alpha inhibitor (55) (100%), non-TNF-alpha inhibitor bDMARD (7) (13%)

						Upadacitinib 12mg twice-daily for 12 weeks	55	59	11	45	1987 ACR Revised Criteria	55	NA	NA	NA	NA	NA	TNF-alpha inhibitor (55) (100%), non-TNF-alpha inhibitor bDMARD (14) (26%)
						Upadacitinib 6mg twice-daily for 12 weeks	55	56	12	43	1987 ACR Revised Criteria	55	NA	NA	NA	NA	NA	TNF-alpha inhibitor (55) (100%), non-TNF-alpha inhibitor bDMARD (14) (26%)
						Upadacitinib 3mg twice-daily for 12 weeks	55	57	13	43	1987 ACR Revised Criteria	55	NA	NA	NA	NA	NA	TNF-alpha inhibitor (55) (100%), non-TNF-alpha inhibitor bDMARD (10) (18%)
						Placebo twice-daily for 12 weeks	56	58	12	48	1987 ACR Revised Criteria	56	NA	NA	NA	NA	NA	TNF-alpha inhibitor (55) (100%), non-TNF-alpha inhibitor bDMARD (9) (16%)
Fleischmann et al.	2022	NCT03682705	Upadacitinib	JAK1 inhibitor	12	Upadacitinib 15mg once-daily for 12 weeks	40	57.7	10.6	35	2010 ACR and EULAR	20	5	NA	NA	NA	NA	bDMARDs (40) (100%)
						Placebo once-daily for 12 weeks	19	57.6	9.1	17	2010 ACR and EULAR	8	3	NA	NA	NA	NA	bDMARDs (19) (100%)
Genovese et al.	2017	NCT01565655	Peficitinib	JAK1/2 inhibitor	12	Peficitinib 150mg once-daily for 12 weeks	64	54.4	12.5	50	1987 ACR Revised Criteria	0	0	6 (9.4%)	0	4 (6.3%)	12	MTX (49) (76.6%), LFN (10) (15.6%), bDMARD (33) (51.6%)
						Peficitinib 100mg once-daily for 12 weeks	58	54.9	11.3	51	1987 ACR Revised Criteria	0	0	5 (8.6%)	0	7 (12.1%)	13	MTX (41) (70.7%), LFN (9) (15.5%), bDMARD (25) (43.1%)
						Peficitinib 50mg once-daily for 12 weeks	57	54.8	10	48	1987 ACR Revised Criteria	0	0	1 (1.8%)	0	5 (8.8%)	21	MTX (36) (63.2%), LFN (10) (17.5%), bDMARD (28) (49.1%)
						Peficitinib 25mg once-daily for 12 weeks	59	52.6	10.2	48	1987 ACR Revised Criteria	0	0	5 (8.5%)	0	9 (15.3%)	12	MTX (41) (69.5%), LFN (10) (16.9%), bDMARD (27) (45.8%)
						Placebo once-daily for 12 weeks	51	52.7	12.2	42	1987 ACR Revised Criteria	0	0	10 (19.6%)	0	4 (7.8%)	9	MTX (35) (68.6%), LFN (8) (15.7%), bDMARD (27) (52.9%)
Tanaka et al.	2018		Upadacitinib	JAK1 inhibitor	12	Upadacitinib 30mg once-daily for 12 weeks	50											
						Upadacitinib 15mg once-daily for 12 weeks	49											

						Upadacitinib 7.5mg once-daily for 12 weeks	49											
						Placebo once-daily for 12 weeks	49											
Tanaka et al.	2011	NCT00 603512	Tofacitinib	JAK inhibitor	12	Tofacitinib 10mg twice-daily for 12 weeks	26	50.6	10	25	1987 ACR Revised Criteria	26	NA	NA	NA	NA	13	NA
						Tofacitinib 5mg twice-daily for 12 weeks	27	50	9.8	22	1987 ACR Revised Criteria	27	NA	NA	NA	NA	15	NA
						Tofacitinib 3mg twice-daily for 12 weeks	27	53.3	12.1	24	1987 ACR Revised Criteria	27	NA	NA	NA	NA	17	NA
						Tofacitinib 1mg twice-daily for 12 weeks	28	52	9.4	21	1987 ACR Revised Criteria	28	NA	NA	NA	NA	16	NA
						Placebo twice-daily for 12 weeks	28	50.6	12.4	25	1987 ACR Revised Criteria	28	NA	NA	NA	NA	20	NA

*Refers to the duration of study until primary study outcome assessment occurred

Abbreviations: ACR: American College of Rheumatology; bDMARD: biologic disease-modifying antirheumatic drug; csDMARD: conventional synthetic disease-modifying antirheumatic drug; DMARD: disease modifying antirheumatic drug; EULAR: European League Against Rheumatism; HCQ: hydroxychloroquine; JAK: Janus kinase; LFN: leflunomide; mg: milligram; MOA: mechanism of action; MTX: methotrexate; NA: not available/applicable; NCT: National Clinical Trial; po: per os; RA: rheumatoid arthritis; SD: standard deviation.
