# Supplementary material for: Cumulative incidence and risk of infection in patients with rheumatoid arthritis treated with janus kinase inhibitors: A systematic review and meta-analysis
Source: PLoS One. 2024 Jul 31;19(7):e0306548. doi: 10.1371/journal.pone.0306548 (PMC11290652; doi:10.1371/journal.pone.0306548)
Supplement: S2 Table — (PDF) [file pone.0306548.s014.pdf]

S2 Table. Number and type of infections in patients with RA treated with JAKi from study initiation until primary study outcome assessment, compared to patients in the control group.

Study author	Study Year	Treatment (dose)	Intervention arm			Control arm		
			Non-severe infection (N)	Severe Infection (N)	Opportunistic infection (N)	Non-severe infection (N)	Severe Infection (N)	Opportunistic infection (N)
Genovese et al	2018	Upadacitinib (15mg)	bronchitis (7), gastroenteritis (1), nasopharyngitis (7), pneumonia (1), sinusitis (4), URTIs (13), UTI (16), herpes zoster (2), oral candidiasis (1)	viral infection (1)	herpes zoster (2)	bronchitis (4), gastroenteritis (1), herpes zoster (1), nasopharyngitis (11), pharyngitis (1), sinusitis (2), URTIs (13), UTI (10)	0	herpes zoster (1)
		Upadacitinib (30mg)	bronchitis (4), herpes zoster (2), nasopharyngitis (9), pharyngitis (2), pneumonia (1), sinusitis (1), URTIs (11), UTI (9), oral Candidiasis (1), mucocutaneous candidiasis (1)	herpes zoster (1), ophthalmic herpes zoster (1), pneumonia (2)	herpes zoster (3), ophthalmic herpes zoster (1)			
Zeng et al	2021	Upadacitinib (15mg)	URTI (16), herpes zoster (2)	Herpes zoster (1), pneumonia (3)	Herpes zoster (3)	URTI (11), herpes zoster (1)	Pneumonia (1)	Herpes zoster (1)
Burmester et al	2018	Upadacitinib (30mg)	Bronchitis (5), herpes zoster (1), influenza (3), nasopharyngitis (13), pharyngitis (2), sinusitis (1), URTI (12), UTI (6), oral candidiasis (3)	Varicella infection (1), viral URTI (1), staphylococcal wound infection (1)	varicella infection (1), herpes zoster (1)	Bronchitis (5), herpes zoster (1), influenza (2), nasopharyngitis (9), sinusitis (1), URTI (9), UTI (8), oral candidiasis (1)	Pneumonia (1)	herpes zoster (1)
		Upadacitinib (15mg)	Bronchitis (4), herpes zoster (1), influenza (1), nasopharyngitis (12), pharyngitis (2), sinusitis (6), URTI (12), UTI (8)	Infectious enterocolitis (1)	herpes zoster (1)			
Kameda et al	2020	Upadacitinib (30mg)	Bronchitis (1), gastroenteritis (1), herpes zoster (2), nasopharyngitis (9), tinea pedis (1), URTI (1)	Hand-foot-and-mouth disease (1), herpes zoster (1), *P. jirovecii* pneumonia (1)	herpes zoster (3), *Pneumocystis jirovecii* pneumonia (1)	Herpes zoster (1), influenza (2), nasopharyngitis (5)	0	herpes zoster (1)
		Upadacitinib (15mg)	Bronchitis (1), cystitis (1), gastroenteritis (1), nasopharyngitis (6), URTI (1)	Cellulitis (1)				
		Upadacitinib (7.5mg)	Bronchitis (1), herpes zoster (1), nasopharyngitis (5), oral herpes (2), pharyngitis (2)	0	herpes zoster (1)			
Genovese et al	2016	Upadacitinib (24mg)	Nasopharyngitis (3), herpes zoster (2)	0	herpes zoster (2)	influenza (1)	0	0
		Upadacitinib (18mg)	Nasopharyngitis (2), influenza (1)	0	0			
		Upadacitinib (12mg)	Nasopharyngitis (4), influenza (4)	Pneumonia (1)	0			
		Upadacitinib (6mg)	influenza (2)	0	0			
		Upadacitinib (3mg)	influenza (1), herpes zoster (1)	0	herpes zoster (1)			
Kremer et al	2015	Upadacitinib (18mg)	Gastroenteritis (3), sinusitis (1), URTI (4), UTI (7), herpes zoster (1)	0	herpes zoster (1)	Gastroenteritis (1), sinusitis (3), URTI (1), UTI (2), herpes zoster (2)	0	herpes zoster (2)
		Upadacitinib (12mg)	Sinusitis (1), URTI (4), UTI (2), oral candidiasis (1), herpes zoster (1)	0	herpes zoster (1)			
		Upadacitinib (6mg)	URTI (1), UTI (1)	0	0			
		Upadacitinib (3mg)	Sinusitis (3), URTI (2), UTI (2), herpes zoster (1)	0	herpes zoster (1)			
Fleischmann et al	2022	Upadacitinib (15mg)	Tooth infection (1), URTI (2), UTI (3), herpes zoster (1)	0	herpes zoster (1)	Bronchitis (1), sinusitis (1), tooth infection (1), URTI (1)	0	0
Tanaka et al	2018	Upadacitinib (30mg)	total (19), herpes zoster (2)	herpes zoster (1), hand-foot-mouth-disease (1), *P. jirovecii* pneumonia (1)	herpes zoster (3), *P. jirovecii* pneumonia (1)	total (11), herpes zoster (1)	0	herpes zoster (1)
		Upadacitinib (15mg)	15	1 (cellulitis)	0			
		Upadacitinib (7.5mg)	18 (herpes zoster 1)	0	herpes zoster (1)			
Fleischmann et al	2017	Baricitinib (4mg)	Herpes zoster (5), nasopharyngitis (21), pharyngitis (7), URTIs (16), bronchitis (9), gastroenteritis (6), influenza (11), rhinitis (2), sinusitis (7), UTI (14), vulvovaginal candidiasis (6)	Acute hepatitis B (1), Campylobacter gastroenteritis (1), P. jirovecii pneumonia (1), pneumonia (1), acute pyelonephritis (1)	*P. jirovecii pneumonia* (1), herpes zoster (5)	Nasopharyngitis (17), pharyngitis (4), URTIs (15), bronchitis (4), gastroenteritis (4), influenza (4), rhinitis (7), sinusitis (5), UTI (7), vulvovaginal candidiasis (1)	Cellulitis (1), *E.coli* sepsis (1), herpes zoster (2), lung infection (1), bacterial meningitis (1), pneumonia (1), sepsis (1), UTI (1)	herpes zoster (2)
Taylor et al	2017	Baricitinib (4mg)	176 in total, Bronchitis (19), influenza (12), nasopharyngitis (37), pharyngitis (12), URTI (15), UTI (21), herpes zoster (5)	Cellulitis (2), epiglottitis (1), herpes zoster (2), pneumonia (1),	herpes zoster (7)	134 in total, Bronchitis (14), influenza (4), nasopharyngitis (35), pharyngitis (14), URTI (14), UTI (16), herpes zoster (2)	Bronchitis (1), gastroenteritis (2), kidney infection (1), muscle abscess (1), pyelonephritis (1), UTI (1)	herpes zoster (2)

Genovese et al	2016	Baricitinib (4mg)	Bronchitis (10), gastroenteritis (6), herpes zoster (6), influenza (8), nasopharyngitis (9), pharyngitis (5), sinusitis (4), URTI (9), UTI (8)	Gastroenteritis (1), viral gastroenteritis (1), herpes zoster (1), URTI (1), UTI (2), vulval abscess (1)	herpes zoster (7)	Bronchitis (6), gastroenteritis (3), herpes zoster (1), influenza (2), nasopharyngitis (7), rhinitis (1), sinusitis (1), URTI (8), UTI (6)	Cellulitis (2), herpes zoster (1), pneumonia (1), tooth infection (1)	herpes zoster (2)
		Baricitinib (2mg)	Bronchitis (6), gastroenteritis (4), herpes zoster (2), influenza (4), nasopharyngitis (12), pharyngitis (4), rhinitis (5), sinusitis (8), URTI (15), UTI (7), vulvovaginal candidiasis (3)	Campylobacter gastroenteritis (1), intervertebral discitis (1), osteomyelitis (1), pneumonia (1), URTI (1)	herpes zoster (2)			
Dougados et al	2016	Baricitinib (4mg)	Bronchitis (7), gastroenteritis (9), nasopharyngitis (18), pharyngitis (8), sinusitis (4), URTI (24), UTI (9), herpes zoster (3)	Bacterial infection (1), disseminated TB (1), LRTI (1), pneumonia (1), sepsis (1), viral infection (1)	disseminated TB (1), herpes zoster (3)	Bronchitis (11), gastroenteritis (1), nasopharyngitis (18), pharyngitis (3), sinusitis (6), URTI (18), UTI (4)	Bronchitis (1), pneumonia (2), UTI (1), staphylococcal wound infection (1)	0
		Baricitinib (2mg)	Bronchitis (6), gastroenteritis (4), nasopharyngitis (10), pharyngitis (6), sinusitis (3), URTI (14), UTI (12), herpes zoster (4)	Gastroenteritis (1), pneumonia (1)	herpes zoster (4)			
Keystone et al	2014	Baricitinib (8mg)	Bronchitis (1), nasopharyngitis (1), URTI (1), UTI (2)	0	0	Bronchitis (3), nasopharyngitis (2), URTI (2), UTI (4)	0	0
		Baricitinib (4mg)	Bronchitis (2), nasopharyngitis (2), pharyngitis (3), URTI (1), UTI (2)	*Helicobacter* gastritis (1)	0			
		Baricitinib (2mg)	Bronchitis (1), pharyngitis (1), URTI (1), UTI (2)	Bronchitis (1), pneumonia (1)	0			
		Baricitinib (1mg)	Bronchitis (1), pharyngitis (1), UTI (2)	0	0			
Tanaka et al	2016	Baricitinib (8mg)	Nasopharyngitis (2), oral candidiasis (1), pharyngitis (2),	0	0	Influenza (1), nasopharyngitis (6), pharyngitis (1), URTI (1), UTI (1)	0	0
		Baricitinib (4mg)	Folliculitis (1), gingivitis (1), influenza (1), nasopharyngitis (2), pertussis (2)	0	0			
		Baricitinib (2mg)	Cystitis (1), nasopharyngitis (2), pharyngitis (1)	0	0			
		Baricitinib (1mg)	Chronic sinusitis (1), infectious enteritis (1), herpes simplex (1), influenza (1), nasopharyngitis (2), pharyngitis (1), URTI (1)	0	0			
Li et al	2020	Baricitinib	Nasopharyngitis (10), URTI (28), UTI (13)	Diverticulitis (1), herpes zoster (1)	herpes zoster (1)	Nasopharyngitis (6), URTI (22), UTI (5)		
Westhovens et al	2021	Filgotinib (200mg)	Bronchitis (12), nasopharyngitis (21), URTI (42), UTI (19), esophageal candidiasis (1), herpes zoster (6)	LRTI (1), pneumonia (4)	esophageal candidiasis (1), herpes zoster (6)	Bronchitis (15), nasopharyngitis (25), URTI (34), UTI (11), herpes zoster (4)	Abdominal hernia infection (1), appendicitis (1), bronchitis (1), *P. jirovecii* pneumonia (1), pneumonia (1), bacterial pneumonia (1), cryptococcal pneumonia (1), sepsis (1), skin infection (1)	*P. jirovecii* pneumonia (1), cryptococcal pneumonia (1), herpes zoster (4)
		Filgotinib (100mg)	Bronchitis (11), nasopharyngitis (17), URTI (9), UTI (13), herpes zoster (3)	Infective arthritis (1), pneumonia (1), pulmonary sepsis (1), pyelonephritis (1), pyelonephrosis (1) septic shock (1)	Herpes zoster (3)			
Combe e al	2021	Filgotinib (100mg)	133	8	herpes zoster (2)	105	4	herpes zoster (2)
		Filgotinib (200mg)	128	8	herpes zoster (2)			
Genovese et al	2020	Filgotinib (200mg)	Bronchitis (8), nasopharyngitis (15), URTI (8), herpes zoster (1)	Cellulitis (1)	Herpes zoster (1)	Bronchitis (8), nasopharyngitis (7), URTI (6)	Gastroenteritis (2)	0
		Filgotinib (100mg)	Bronchitis (3), nasopharyngitis (9), URTI (9), herpes zoster (2)	Oral abscess (1), bronchitis (1), gallbladder empyema (1), vulval abscess (1)	Herpes zoster (2)			
Westhovens et al	2016	Filgotinib (200mg)	Gastroenteritis (3), nasopharyngitis (7), URTI (6), herpes zoster (3)	Pneumonia (1), erysipelas (1), intervertebral discitis (1), septic shock (1)	herpes zoster (3)	Nasopharyngitis (4), URTI (1), herpes zoster (1)	Appendicitis (1)	herpes zoster (1)
		Filgotinib (100mg)	Gastroenteritis (3), nasopharyngitis (5), URTI (8)	Pneumonia (1), diabetic gangrene (1), subcutaneous abscess (1)	0			

		Filgotinib (50mg)	Gastroenteritis (6), nasopharyngitis (12), URTI (6), herpes zoster (1)	0	herpes zoster (1)			
Kavanaugh et al	2016	Filgotinib (200mg)	NA	Pneumonia (1)	NA	NA	0	0
		Filgotinib (100mg)	NA	0	NA			
		Filgotinib (50mg)	NA	Gastroenteritis (1)	NA			
Kremer et al	2013	Tofacitinib (5mg)	Bronchitis (6), nasopharyngitis (16), pharyngitis (3), URTI (19)	Bronchitis (1), herpes zoster disseminated (1)	herpes zoster disseminated (1)	Bronchitis (4), nasopharyngitis (12), pharyngitis (5), URTI (7)	0	0
		Tofacitinib (10mg)	Bronchitis (3), nasopharyngitis (7), pharyngitis (5), URTI (23)	Diabetic foot infection (1), pneumonia (2)	0			
Kremer et al	2015	Tofacitinib (10mg)	Gastroenteritis (1), nasopharyngitis (8), oral herpes (2), URTI (1), UTI (3)	Bronchopneumonia (1)	0	Gastroenteritis (1), nasopharyngitis (3), URTI (1), UTI (2)	0	0
van Vollenhoven et al	2013	Tofacitinib (10mg)	Bronchitis (3), herpes zoster (5), nasopharyngitis (4), URTI (7), UTI (2)	Bacterial arthritis (1), cellulitis (1), herpes zoster (1), labyrinthitis (1), UTI (1)	herpes zoster (6)	Bronchitis (1), URTI (1)	Sialadenitis (1)	0
		Tofacitinib (5mg)	Bronchitis (2), nasopharyngitis (8), URTI (9), UTI (5)	Osteomyelitis (1), tooth abscess (1), cellulitis (2)	0			
Strand et al	2012	Tofacitinib (10mg)	Influenza (4), nasopharyngitis (5), URTI (8), UTI (10)	Liver abscess (1)	0	Influenza (4), nasopharyngitis (2), URTI (6), UTI (3)	0	0
		Tofacitinib (5mg)	Influenza (2), nasopharyngitis (4), URTI (11), UTI (4)	0	0			
Burmester et al	2013	Tofacitinib (10mg)	Nasopharyngitis (6), sinusitis (1), URTI (2), UTI (3)	0	0	Nasopharyngitis (4), sinusitis (4), URTI (4), UTI (3)	Gastroenteritis (1)	0
		Tofacitinib (5mg)	Nasopharyngitis (5), sinusitis (1), URTI (5), UTI (5)	0	0			
van der Heijde et al	2013	Tofacitinib (10mg)	Nasopharyngitis (13), URTI (7), UTI (3), herpes zoster (5)	influenza (1)	herpes zoster (5),	Nasopharyngitis (1), URTI (5), UTI (4)	0	0
		Tofacitinib (5mg)	Nasopharyngitis (14), URTI (9), UTI (9), herpes zoster (3)	Dengue fever (1), pneumonia (1), viral pneumonia (1)	herpes zoster (3)			
Kremer et al	2009	Tofacitinib (30mg)	Gastroenteritis (1), herpes simplex (2), influenza (4), nasopharyngitis (1), tinea pedis (1), URTI (1), UTI (5)	Acute pyelonephritis (1), Whipple's disease (1)	0	Gastroenteritis (1), influenza (2), nasopharyngitis (1), sinusitis (3), URTI (4), UTI (2), herpes zoster (1)	Staphylococcal pneumonia (1)	Herpes zoster (1)
		Tofacitinib (15mg)	Gastroenteritis (2), herpes simplex (2), influenza (2), nasopharyngitis (1), tinea pedis (3), URTI (3)	Gastroenteritis (1)	0			
		Tofacitinib (5mg)	Gastroenteritis (2), influenza (3), nasopharyngitis (3), tinea versicolor (2), UTI (1), herpes zoster (1)	0	Herpes zoster (1)			
Tanaka et al	2014	Tofacitinib (15mg)	Herpes zoster (1), nasopharyngitis (8), pharyngitis (2)	Herpes zoster oticus (1)	Herpes zoster oticus (1), herpes zoster (1)	Nasopharyngitis (6), pharyngitis (1)	0	0
		Tofacitinib (10mg)	Bronchitis (2), herpes zoster (2), nasopharyngitis (3), pharyngitis (3), URTI (3)	Herpes zoster (1)	Herpes zoster (3)			
		Tofacitinib (5mg)	Nasopharyngitis (6), URTI (1)	Herpes zoster (1)	Herpes zoster (1)			
		Tofacitinib (3mg)	Bronchitis (1), nasopharyngitis (4)	0	0			
		Tofacitinib (1mg)	Bronchitis (1), nasopharyngitis (6)	0	0			
Boyle et al	2014	Tofacitinib (10mg)	Folliculitis (1), herpes zoster (1)	0	herpes zoster (1)	Herpes simplex (1), UTI (1), URTI (1), viral infection (1)	0	0
Tanaka et al	2011	Tofacitinib (10mg)	Cystitis (1), erysipelas (1), gastroenteritis (1), viral gastroenteritis (1), localized infection (1), nasopharyngitis (4), pharyngitis (2), pneumonia (1), viral URTI (1), vulvovaginal candidiasis (1)	0	0	Gastroenteritis (1), nasopharyngitis (4), paronychia (1), tinea pedis (1), URTI (1)	0	0

		Tofacitinib (5mg)	Bronchitis (1), cystitis (1), nasopharyngitis (1), pneumonia (1)	0	0			
		Tofacitinib (3mg)	Acute tonsilitis (1), infected dermatitis (1), viral enterocolitis (1), gastroenteritis (2), nasopharyngitis (1), URTI (1), trichomonas vulvovaginitis (1)	0	0			
		Tofacitinib (1mg)	Nasopharyngitis (3)	0	0			
Takeuchi et al	2019	Peficitinib (150mg)	Bronchitis (3), cystitis (2), gastroenteritis (2), influenza (2), nasopharyngitis (16), pharyngitis (6), URTI (3)	Pyelonephritis (1)	0	cystitis (2), nasopharyngitis (14), pharyngitis (6), URTI (4)	0	0
		Peficitinib (100mg)	Bronchitis (3), cystitis (1), gastroenteritis (1), influenza (1), nasopharyngitis (19), pharyngitis (3), URTI (3)	Herpes zoster (1), *P. jirovecii* pneumonia (1), pneumonia (1)	Herpes zoster (1), *P. jirovecii* pneumonia (1)			
Tanaka et al	2019	Peficitinib (150mg)	Bronchitis (1), gastroenteritis (1), influenza (1), nasopharyngitis (19), pharyngitis (1), URTI (3)	*P. jirovecii* pneumonia (1)	*P. jirovecii* pneumonia (1)	Bronchitis (1), influenza (2), nasopharyngitis (6), pharyngitis (1), URTI (2)	0	0
		Peficitinib (100mg)	Bronchitis (4), herpes zoster (1), influenza (3), nasopharyngitis (10), pharyngitis (1), URTI (3)	0	herpes zoster (1)			
Takeuchi et al	2015	Peficitinib (150mg)	17	0	0	12	0	0
		Peficitinib (100mg)	7	0	Herpes zoster (2)			
		Peficitinib (50mg)	14	0	0			
		Peficitinib (25mg)	18	0	Herpes zoster (2)			
Kivitz et al	2017	Peficitinib (150mg)	NA	erysipelas (1)	herpes zoster (1)	NA	0	0
		Peficitinib (100mg)	NA	viral infection (1)	herpes zoster (2)			
		Peficitinib (50mg)	NA	0	0			
		Peficitinib (25mg)	NA	0	0			
Genovese et al	2017	Peficitinib (150mg)	NA	0	0	NA	0	herpes zoster (1)
		Peficitinib (100mg)	NA	0	0			
		Peficitinib (50mg)	NA	0	0			
		Peficitinib (25mg)	NA	limb abscess (1)	0			

Abbreviations: JAKi: Janus-activated kinase inhibitor; RA: rheumatoid arthritis; TB: tuberculosis; URTI: upper respiratory tract infection; UTI: urinary tract infection
