# Supplementary material for: Cumulative incidence and risk of infection in patients with rheumatoid arthritis treated with janus kinase inhibitors: A systematic review and meta-analysis
Source: PLoS One. 2024 Jul 31;19(7):e0306548. doi: 10.1371/journal.pone.0306548 (PMC11290652; doi:10.1371/journal.pone.0306548)
Supplement: S3 Table — (PDF) [file pone.0306548.s015.pdf]

S3 Table. Number and type of infections in patients with RA treated with JAKi during follow-up, extending from the time of primary study outcome assessment until the end of the study, compared to patients in the control group.

			Intervention arm			
**Study author**	**Study Year**	**Follow-up weeks**	**Treatment (dose)**	**Non-severe infection (N)**	**Severe Infection (N)**	**Opportunistic infection (N)**
Zeng et al.	2021	Weeks 12-64	Upadacitinib	URTI (38), herpes zoster (23), latent TB (18), nasopharyngitis (24), UTI (19)	Herpes zoster (3), pneumonia (4), anal abscess (1), appendicitis (2), bacterial infection (1), fallopian tube abscess (1), febrile infection (1), influenza (1), lung infection (1), oral herpes (1), otitis media (1), cryptococcal pneumonia (1), post-op wound infection (2), fungal sinusitis (1)	cryptococcal pneumonia (1), herpes zoster (26)
Kameda et al.	2020	Weeks 12-60	Upadacitinib (30mg)	Bronchitis (3), cystitis (4), gastroenteritis (4), herpes zoster (7), influenza (4), nasopharyngitis (15), oral herpes (3), pharyngitis (5), tinea pedis (6), URTI (3)	Herpes zoster (2), *Pneumocystis jirovecii* pneumonia (3), pneumonia (2), bacterial pneumonia (1)	Herpes zoster (9), *P. jirovecii* pneumonia (3)
			Upadacitinib (15mg)	Bronchitis (3), cystitis (3), gastroenteritis (3), herpes zoster (8), influenza (7), nasopharyngitis (24), oral herpes (1), pharyngitis (5), tine pedis (4), URTI (1)	Bacterial infection (1), herpes zoster (1), bacterial pneumonia (1), acute pyelonephritis (1), rhinitis (1)	herpes zoster (9)
			Upadacitinib (7.5mg)	Bronchitis (6), gastroenteritis (3), herpes zoster (3), influenza (6), nasopharyngitis (25), oral herpes (3), pharyngitis (4), URTI (2)	Appendicitis (1), herpes zoster (5), pneumonia (1)	herpes zoster (2)
Taylor et al.	2017	Weeks 24-52	Baricitinib	Bronchitis (33), influenza (13), nasopharyngitis (39), pharyngitis (14), URTI (23), UTI (15)	Cellulitis (1), herpes zoster (1), pneumonia (1), UTI (2), atypical pneumonia (1), bacteremia (1), cystitis (1), necrotizing fasciitis (1), acute pyelonephritis (1), viral infection (1),	herpes zoster (1)
Keystone et al.	2014	Weeks 12-24	Baricitinib (8mg)	Nasopharyngitis (2), URTI (2), UTI (2)	Helicobacter gastritis (1), bacterial pneumonia (1)	0
			Baricitinib (4mg)	Bronchitis (2), URTI (5), UTI (2)	0	0
			Baricitinib (2mg)	Bronchitis (3), nasopharyngitis (1), pharyngitis (2), URTI (4), UTI (3)	0	0
Tanaka et al.	2016	Weeks 12-52	Baricitinib (4mg)	Acute tonsilitis (3), bacteriuria (1), bronchitis (3), cellulitis (1), cystitis (1), eczema impetiginous (1), bacterial enterocolitis (1), gastroenteritis (1), norovirus gastroenteritis (1), viral gastroenteritis (1), Helicobacter infection (1), herpes virus infection (1), herpes zoster (4), influenza (2), molloscum contagiosum (1), nasopharyngitis (22), oral candidiasis (1), oral herpes (2), paronychia (1), periodontitis (1), pharyngitis (3), skin infection (1), tinea pedis (2), tonsilitis (1), URTI (3), UTI (1), viral infection (1), vulval abscess (1)	Gastroenteritis (1), Herpes zoster (1), *P. jirovecii* pneumonia (1), pneumonia (1)	Herpes zoster (5), *P. jirovecii* pneumonia (1)
			Baricitinib (8mg)	Appendicitis (1), bacteriuria (1), bronchitis (2), cystitis (5), diverticulitis (1), empyema (1), infectious enterocolitis (1), fungal paronychia (1), fungal skin infection (1), furuncle (1), gastroenteritis (3), genital herpes (1), gingivitis (1), Helicobacter infection (1), herpes zoster (4), influenza (3), nasopharyngitis (16), oropharyngeal candidiasis (1), oral herpes (1), paronychia (2), periodontitis (1), pharyngitis (3), pneumonia (1), dental pulpitis (2), rhinitis (1), sinusitis (2), candida skin (1), tinea pedis (1), tinea versicolor (1), URTI (3), viral rash (1)	Herpes zoster (2), *P. jirovecii* pneumonia (1)	Herpes zoster (6), *P. jirovecii* pneumonia (1)
Li et al.	2020	Weeks 24-52	Baricitinib (4mg or 2mg)	Bronchitis (5), gastroenteritis (3), influenza (3), nasopharyngitis (8), URTI (16), UTI (7)	Herpes zoster (1), lung infection (1), pneumonia (1), soft tissue infection (1), viral infection (1)	herpes zoster (1)

Combe et al.	2021	Weeks 24-52	Filgotinib (200mg)	206, nasopharyngitis (43), URTI (41), UTI (18), herpes zoster (6)	limb abscess (1), infective arthritis (1), bronchitis (2), cellulitis (1), gastroenteritis (1), paronychia (1), pneumonia (4), viral pneumonia (1), septic shock (2), UTI (1)	6 (herpes zoster)
			Filgotinib (100mg)	194, nasopharyngitis (48), URTI (49), UTI (19), herpes zoster (4)	Candida infection (1), cellulitis (1), erysipelas (1), infected skin ulcer (1), osteomyelitis (1), pneumonia (4), bacterial pneumonia (1), acute pyelonephritis (1), sinusitis (1), tooth abscess (1), UTI (1), varicella (1)	Herpes zoster (4)
Kavanaugh et al.	2016	Weeks 12-24	Filgotinib (200mg)	URTI (4), UTI (4)	0	0
			Filgotinib (100mg)	URTI (4), UTI (7)	Cellulitis (1), chronic pyelonephritis (1)	0
			Filgotinib (50mg)	URTI (2), UTI (3), herpes zoster (1)	0	herpes zoster (1)
Kremer et al.	2013	Weeks 12-48	Tofacitinib (5mg)	Bronchitis (6), Nasopharyngitis (6), pharyngitis (1), URTI (24), gastroenteritis (7), sinusitis (7), UTI (8)	0	0
			Tofacitinib (10mg)	Bronchitis (10), nasopharyngitis (9), pharyngitis (2), URTI (27), gastroenteritis (2), sinusitis (3), UTI (12)	Bronchiectasis (1), Pulmonary TB (1)	Pulmonary TB (1)
van Vollenhoven et al.	2013	Weeks 12-24	Tofacitinib (10mg)	Nasopharyngitis (2), URTI (1), UTI (1)	clostridial infection (1)	0
			Tofacitinib (5mg)	Nasopharyngitis (5), URTI (5), UTI (1)	Cellulitis (1), gastroenteritis (1), localized infection (1), septic shock (1)	0
Strand et al.	2012	Weeks 12-24	Tofacitinib (10mg)	Influenza (6), URTI (8), UTI (11)	Bronchitis (1), pyelonephritis (1), TB pleurisy (1)	TB pleurisy (1)
			Tofacitinib (5mg)	Influenza (5), URTI (14), UTI (3)	Cellulitis (2)	0
Burmester et al.	2013	Weeks 12-24	Tofacitinib (10mg)	Bronchitis (3), nasopharyngitis (5), sinusitis (4), URTI (8), UTI (2)	Diverticulitis (1), pyelonephritis (1)	0
			Tofacitinib (5mg)	Bronchitis (6), nasopharyngitis (6), sinusitis (1), URTI (5), UTI (4)	Bronchopneumonia (1)	0
van der Heijde	2013	Weeks 12-24	Tofacitinib (10mg)	Nasopharyngitis (9), URTI (9), bronchitis (9), herpes zoster (4)	Bacterial enterocolitis (1), gastroenteritis (1), UTI (1)	herpes zoster (4)
			Tofacitinib (5mg)	Nasopharyngitis (13), URTI (17), bronchitis (3), herpes zoster (4)	Pneumonia (1), cellulitis (2), herpes zoster (2), atypical pneumonia (1), acute pyelonephritis (1), sepsis (1)	herpes zoster (6)
Takeuchi et al.	2019	Weeks 12-52	Peficitinib (150mg)	Bronchitis (9), cystitis (6), gastroenteritis (6), influenza (2), nasopharyngitis (57), pharyngitis (8), URTI (11)	Cellulitis (2), pneumonia (2), cryptococcal pneumonia (1)	cryptococcal pneumonia (1)
			Peficitinib (100mg)	Bronchitis (5), cystitis (6), gastroenteritis (7), influenza (3), nasopharyngitis (45), pharyngitis (9), URTI (7)	Pneumonia (2), pyelonephritis (1)	0
Tanaka et al.	2019	Weeks 12-52	Peficitinib (150mg)	Bronchitis (5), gastroenteritis (2), herpes zoster (5), influenza (6), nasopharyngitis (29), pharyngitis (11), URTI (8)	Pharyngitis (1), pneumonia (1)	herpes zoster (5)
			Peficitinib (100mg)	Bronchitis (4), gastroenteritis (4), herpes zoster (8), influenza (6), nasopharyngitis (34), pharyngitis (7), URTI (7)	Eczema herpeticum (1), pneumococcal pneumonia (1)	herpes zoster (8)

Abbreviations: JAKi: Janus-activated kinase inhibitor; RA: rheumatoid arthritis; TB: tuberculosis; URTI: upper respiratory tract infection; UTI: urinary tract infection
