# Supplementary material for: Cumulative incidence and risk of infection in patients with rheumatoid arthritis treated with janus kinase inhibitors: A systematic review and meta-analysis
Source: PLoS One. 2024 Jul 31;19(7):e0306548. doi: 10.1371/journal.pone.0306548 (PMC11290652; doi:10.1371/journal.pone.0306548)
Supplement: S4 Table — (PDF) [file pone.0306548.s016.pdf]

S4 Table. Random-effects meta-regression analysis for age, sex, current use of MTX and/or corticosteroids and risk of any-grade, severe, or opportunistic infection in patients with RA treated with JAKi compared to patients in the control group.

Risk of any-grade infection in patients receiving any JAKi			
**Covariate**	**Coefficient**	**95% CI**	**p-value**
Age	0.013	-0.038 – 0.064	0.591
Female gender	-1.439	-3.887 – 1.010	0.230
Corticosteroid use	-0.091	-0.606 – 0.423	0.710
MTX use	0.765	-2.568 – 4.098	0.632
Risk of severe infection in patients receiving any JAKi			
**Covariate**	**Coefficient**	**95% CI**	**p-value**
Age	0.099	-0.097 – 0.295	0.301
Female gender	0.482	-8.633 – 9.596	0.912
Corticosteroid use	-0.166	-2.028 – 1.696	0.853
MTX use	-5.423	-17.656 – 6.796	0.360
Risk of opportunistic infections in patients receiving any JAKi			
**Covariate**	**Coefficient**	**95% CI**	**p-value**
Age	0.0136	-0.246 – 0.269	0.911
Female gender	-1.010	-10.058 – 8.038	0.815
Corticosteroid use	1.076	-1.861 – 4.014	0.447
MTX use	-0.288	-16.167 – 15.592	0.970

Abbreviations: CI: confidence interval; MTX: methotrexate; RA: rheumatoid arthritis; JAKi: Janus-activated kinase inhibitor.
