# Supplementary material for: Cumulative incidence and risk of infection in patients with rheumatoid arthritis treated with janus kinase inhibitors: A systematic review and meta-analysis
Source: PLoS One. 2024 Jul 31;19(7):e0306548. doi: 10.1371/journal.pone.0306548 (PMC11290652; doi:10.1371/journal.pone.0306548)
Supplement: S5 Table — (PDF) [file pone.0306548.s017.pdf]

S5 Table. Quality assessment of the included studies using the RoB 2 tool.

Study ID	Experimental	Comparator	Outcome	D1	D2	D3	D4	D5	Overall
Keystone et al. (Baricitinib - NCT01185353)	Baricitinib	Control	Infection	+	+	+	+	+	+
Tanaka et al. (Baricitinib - NCT01469013)	Baricitinib	Control	Infection	+	+	+	+	+	+
Li et al. (Baricitnib - NCT02265705)	Baricitinib	Control	Infection	+	+	+	+	+	+
Genovese et al. (Baricitinib - NCT01721044)	Baricitinib	Control	Infection	+	+	+	+	+	+
Taylor et al. (Baricitinib - NCT01710358)	Baricitinib	Control	Infection	+	+	+	+	+	+
Fleischmann et al. (Baricitinib - NCT01711359)	Baricitinib	Control	Infection	+	+	+	+	+	+
Dougados et al. (Baricitinib - NCT01721057)	Baricitinib	Control	Infection	+	+	+	+	+	+
Westhovens et al. (Filgotinib - NCT01888874)	Filgotinib	Control	Infection	+	+	+	+	+	+
Kavanaugh et al. (Filgotinib - NCT01894516)	Filgotinib	Control	Infection	+	+	+	+	+	+
Combe et al. (Filgotinib - NCT02889796)	Filgotinib	Control	Infection	+	+	+	+	+	+
Genovese et al. (Filgotinib - NCT02873936)	Filgotinib	Control	Infection	+	+	+	+	+	+
Westhovens et al. (Filgotinib - NCT02886728)	Filgotinib	Control	Infection	+	+	+	+	+	+
Kivitz et al. (Peficitinib - NCT01554696)	Peficitinib	Control	Infection	+	+	+	+	+	+
Genovese et al. (Peficitinib - NCT01565655)	Peficitinib	Control	Infection	+	+	+	+	+	+
Takeuchi et al. (Peficitinib - NCT01649999)	Peficitinib	Control	Infection	+	+	+	+	+	+
Tanaka et al. (Peficitinib - NCT02308163)	Peficitinib	Control	Infection	+	+	+	+	+	+
Takeuchi et al. (Peficitinib - NCT02305849)	Peficitinib	Control	Infection	+	+	+	+	+	+
Kremer et al. (Tofacitinib - NCT00147498)	Tofacitinib	Control	Infection	+	+	+	+	+	+
Kremer et al. (Tofacitinib - NCT00413660)	Tofacitinib	Control	Infection	+	+	+	+	+	+
Tanaka et al. (Tofacitinib - NCT00603512)	Tofacitinib	Control	Infection	+	+	+	+	+	+
Tanaka et al. (Tofacitinib - NCT00687193)	Tofacitinib	Control	Infection	+	+	+	+	+	+
Boyle et al. (Tofacitinib - NCT00976599)	Tofacitinib	Control	Infection	+	+	+	+	+	+
Kremer et al. (Tofacitinib - NCT01484561)	Tofacitinib	Control	Infection	+	+	+	+	+	+
van Vollenhoven et al. (Tofacitinib - NCT00853385)	Tofacitinib	Control	Infection	+	+	+	+	+	+
van der Heijde et al. (Tofacitinib - NCT00847613)	Tofacitinib	Control	Infection	+	+	+	+	+	+
Strand et al. (Tofacitinib - NCT00814307)	Tofacitinib	Control	Infection	+	+	+	+	+	+
Burmester et al. (Tofacitinib - NCT00960440)	Tofacitinib	Control	Infection	+	+	+	+	+	+
Kremer et al. (Tofacitinib - NCT008556544)	Tofacitinib	Control	Infection	+	+	+	+	+	+
Tanaka et al. (Upadacitinib)	Upadacitinib	Control	Infection	!	+	+	+	+	+
Kremer et al. (Upadacitinib - NCT01960855)	Upadacitinib	Control	Infection	+	+	+	+	+	+
Genovese et al. (Upadacitinib - NCT02066389)	Upadacitinib	Control	Infection	+	+	+	+	+	+
Zeng et al. (Updacitinib - NCT02955212)	Upadacitinib	Control	Infection	+	+	+	+	+	+
Fleischmann et al. (Upadacitinib - NCT03682705)	Upadacitinib	Control	Infection	+	+	+	+	+	+
Genovese et al. (Upadacitinib - NCT02706847)	Upadacitinib	Control	Infection	+	+	+	+	+	+
Kameda et al. (Upadacitinib - NCT02720523)	Upadacitinib	Control	Infection	+	+	+	+	+	+

RoB 2 domains: D1: randomization process; D2: deviations from intended interventions; D3: missing outcome data; D4: measurement of the outcome; D5: selection of the reported results.
